# Detection of horizontal transfer of individual genes by anomalous oligomer frequencies

**DOI:** 10.1186/1471-2164-13-245

**Published:** 2012-06-15

**Authors:** Jeff Elhai, Hailan Liu, Arnaud Taton

**Affiliations:** 1 Center for the Study of Biological Complexity, Virginia Commonwealth University, Richmond, VA 23284, USA; 2 Institute of Bioinformatics, College of Agriculture and Biotechnology, Zhejiang University, Hangzhou,, Zhejiang 310058, China; 3 Division of Biological Sciences, University of California San Diego, La Jolla, San Diego, CA, 92093, USA

## Abstract

**Background:**

Understanding the history of life requires that we understand the transfer of genetic material across phylogenetic boundaries. Detecting genes that were acquired by means other than vertical descent is a basic step in that process. Detection by discordant phylogenies is computationally expensive and not always definitive. Many have used easily computed compositional features as an alternative procedure. However, different compositional methods produce different predictions, and the effectiveness of any method is not well established.

**Results:**

The ability of octamer frequency comparisons to detect genes artificially seeded in cyanobacterial genomes was markedly increased by using as a training set those genes that are highly conserved over all bacteria. Using a subset of octamer frequencies in such tests also increased effectiveness, but this depended on the specific target genome and the source of the contaminating genes. The presence of high frequency octamers and the GC content of the contaminating genes were important considerations. A method comprising best practices from these tests was devised, the Core Gene Similarity (CGS) method, and it performed better than simple octamer frequency analysis, codon bias, or GC contrasts in detecting seeded genes or naturally occurring transposons. From a comparison of predictions with phylogenetic trees, it appears that the effectiveness of the method is confined to horizontal transfer events that have occurred recently in evolutionary time.

**Conclusions:**

The CGS method may be an improvement over existing surrogate methods to detect genes of foreign origin.

## Background

A significant fraction of genes of many organisms appears to have arisen not vertically, by lineal descent from ancient ancestors of the organisms, but rather horizontally, by acquisition from outside the line [1,2]. Recognition of those genes acquired by horizontal transfer is necessary to reconstruct the evolutionary events that shape genomes and is useful in understanding the mechanisms by which that shaping occurs [1,3,4].

Horizontal transfer may be viewed conceptually as a discordance between the phylogeny of a gene and a reference phylogeny of the cell that contains it, and so the analysis of phylogenetic trees would seem to be a natural tool to detect transfer events [5,6]. The construction of informative trees, however, may be computationally intensive and require sequence information from related species that is often not available. Furthermore, the analysis of trees is by no means straightforward [7,8].

Several surrogate methods have been put forth to identify alien genes in a genome, using the sequence characteristics of the genes rather than phylogenetic information. Specific genes of putative alien origin have been identified through unusual G + C content [9], codon usage [10,11], or G + C content at position 1 and/or 3 within codons [12]. Compositional contrasts, measured through frequencies of longer oligonucleotides or high order Markov models, have been used to predict horizontal transfer [13-17], with oligonucleotides as long as eight or nine found to be more effective than shorter oligonucleotides [13,16].

All of these methods share a family resemblance: a norm is established for a sequence characteristic, and genes or genomic regions are sought that deviate from the norm. For example, Tsirigos and Rigoutsos (2005) [16] determined the frequencies of all octanucleotides in a given genome and then compared these frequencies to those determined for a specific gene, using statistical tests to assess whether the deviation from the first set of frequencies was significant. A few critical components of such tests are evident: The choice of the characteristic, the choice of the norm, and the choice of how to determine significance.

It is important to note that many of these methods have produced strikingly different predictions as to the extent of horizontal transfer [18] and the specific genes predicted to be alien [6], possibly because they are sensitive to different ages of transfer events [19,20] or because they are not all reliable predictors of evolutionary events. Most applications of these methods use the entire genome or the set of all genes within it as a training set, but this practice taints the training set with the foreign DNA and genes that are sought [21].

We have developed a method to detect horizontally transferred genes that seeks to maximize the signal to noise ratio in oligonucleotide contrasts by limiting the training set to conserved core genes [22], thereby (by hypothesis) removing foreign genes. At the same time, we considered that the paucity of genus-associated restriction sites in many genomes [23,24] and the rapidity with which genes encoding restriction enzymes are gained and lost in organisms [25] might point to underrepresented oligonucleotides as a source of a genome signature that changes rapidly over evolutionary time. We therefore initially focused on this subset, hoping to enhance the organism-specific signal.

## Results

### Method of evaluation

A method intended to detect horizontal gene transfer may fail on either of two counts: a failure to detect a genuinely foreign gene (false negative) or an erroneous claim that a genuinely native gene is foreign (false positive). To test for both possibilities, we assessed methods using two sets of genes, those artificially introduced into the genome from a single foreign source (the test-foreign set) and those within the genome that were deemed provisionally to be genuinely native (the test-native set). The latter were identified as those genes having orthologs in all considered cyanobacteria (after removing the core genes, i.e. the fraction used to construct the reference frequency set, as described later).

**Figure 1:**
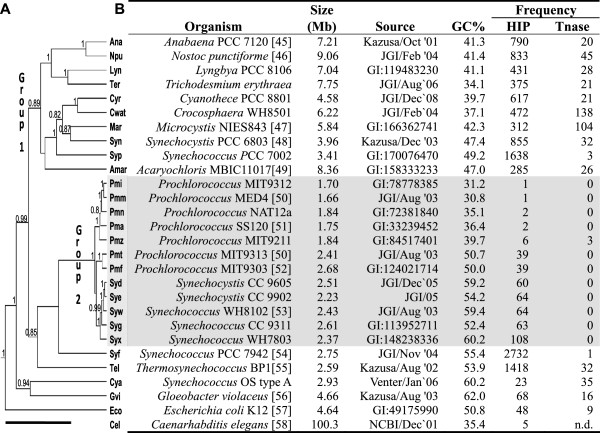
**Characteristics of genomes used in this study.** (**A**) The phylogenetic tree was inferred from 16 S rRNA gene sequences using a Bayesian approach as described in the Methods section. The posterior probabilities are indicated at the nodes when equal to or greater than 80%. The length of the thick line at the bottom represents 0.1 mutations per position. The tree shown is substantially the same as that derived from other methods (Additional file 1). The shading highlights the well isolated clade of small marine *Prochlorococcus* and *Synechococcus* (Group 2). At the end of each leaf is the nickname of the organism used in this study. 3-letter nicknames are those used by KEGG. (**B**) Other characteristics of the genome. HIP1 frequency is given as the number of GCGATCGC sequences per 1 million nucleotides of genome sequence. The transposase (Tn) frequency is given as the number of annotated transposase genes per 1 million nucleotides of genome sequence. The source of the genome sequence is NCBI, with the given accession number, unless otherwise specified. The other sources are Kazusa DNA Research Institute, Joint Genome Institute (JGI), and the J. Craig Venter Institute. Published sources, when available, are given in references [27-40]. n.d. = not determined.

We chose to focus primarily on cyanobacterial genomes, because cyanobacteria constitute a broad taxon with a large number of fully sequenced genomes, and because a tool exists, CyanoBIKE [26], that greatly facilitates analysis of their genomes. With genomes from organisms of varying phylogenetic distance from each other (Figure 1 and Additional file 1), we were able to assess the effect of distance on the ability of different tools to pick out foreign genes.

**Figure 2:**
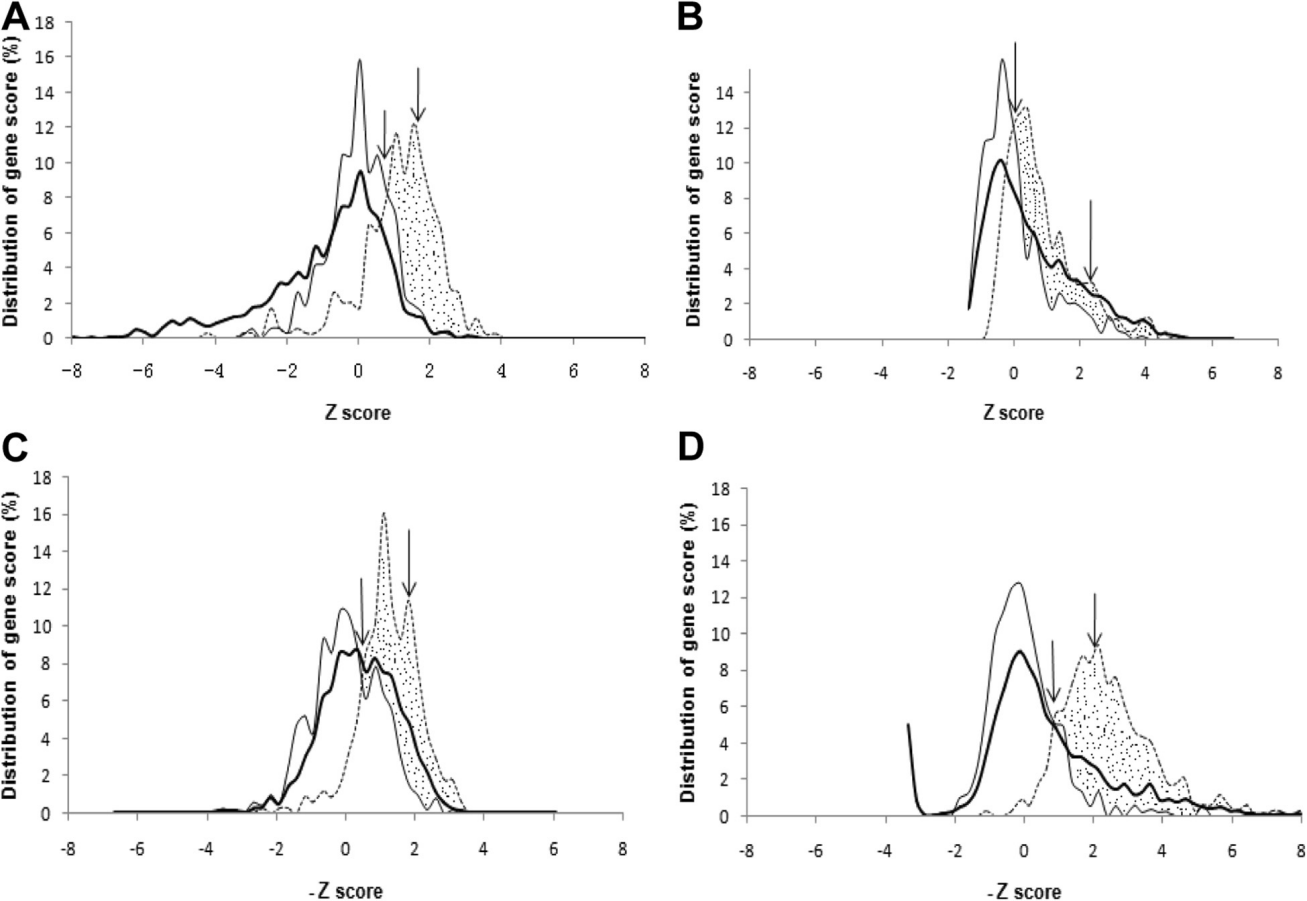
**Distribution of gene scores according to four methods.** Coding genes of *Syn* contaminated with 114 genes (3% of the total number of coding genes of *Syn*) from *Tel* were used. The z-score is the deviation of a score of a gene from the mean score, in units of standard deviations. Z-scores were binned every 0.25 units. For the two scoring methods (W8 and CGS) that use covariance, the signs of the Z scores were reversed so that putative foreign genes would lie on the right side of the graph (see Methods). The thick lines, thin lines, and dashed lines show the distributions of scores for all coding genes, test core genes, and introduced foreign genes, respectively. The right-most arrow identifies the z-score that splits the test core gene distribution into a ratio of 95:5. The left-most arrow identifies the z-score that maximizes the difference between the number of scores of introduced genes and number of scores of test core genes to the right of the arrow, a score that occurs at the intersection of the two curves. The shaded area is the maximal discrimination, i.e., the area under the dashed line minus the area under the thin line (the number of true positives minus the number of false positives) using the threshold marked by the left-most arrow. **(A)** GC, (**B**) Codon bias, (**C**) W8, (**D**) CGS.

The goal of any method is to score genes in such a way as to maximize the separation between native genes and genes of foreign origin. Figure 2 shows examples of the distribution of scores for the four methods considered: G + C frequency (GC), codon bias [11], octanucleotide frequency (W8, as described above and in reference 16), and Core Gene Similarity (CGS, described below). We define here *discrimination* as the difference between the fraction of true positives (estimated by the fraction of the test-foreign set predicted to be foreign at a given threshold) minus the fraction of false-positives (estimated by the fraction of the test-native set predicted to be foreign at the same threshold). The decision of where to place a threshold value is ultimately a matter of choice and will be discussed later. For testing purposes, we determined the *maximal discrimination*, defined as the discrimination at the threshold value that maximizes the difference between the detection of test-foreign and test-native genes. This is equivalent to finding the x-value that produces the maximum area under the curve, as shown in Figure 2.

### Tests of assumptions

The motivation behind this work stems from two assumptions. The first is that methods that compare the characteristics of individual genes to those derived from an entire genome will suffer because the genome (by hypothesis) is contaminated by genes of foreign origin. The second assumption is that foreign genes will be more easily discerned if oligonucleotide frequency comparisons are confined to those frequencies that are the most informative. Including other frequencies in the analysis will only reduce the signal to noise ratio. Each assumption was tested under controlled albeit artificial conditions.

**Figure 3:**
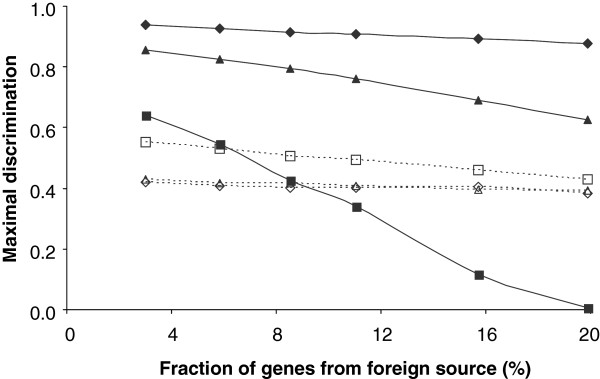
**Degradation of maximal discrimination by increasing contamination of a genome with foreign DNA.** The genome of *Syn* was contaminated with genes from either *Pmm* (solid symbols) or *Tel* (hollow symbols), measuring maximal discrimination either by W8 (□,■), codon bias (◊,♦), or GC% (∆,▲). Maximal discrimination measured by CGS is not shown because its reference set (hence its calculated scores) is not affected by contaminating foreign genes.

To determine the effect on the methods by contamination of a genome by foreign genes, the genome of *Synechocystis* PCC 6803 (*Syn*; see Figure 1 for abbreviations) was seeded with varying numbers of genes from two organisms, One organism, *Prochlorococcus* sp. MED4 (*Pmm*), was chosen because it is evolutionarily distant from *Syn* and has a very different GC content (31% compared to 47%), and a second, *Thermosynechococcus elongatus* BP1 (*Tel*), was chosen as one that is more similar to *Syn* with respect to GC fraction (though still phylogenetically very distant) and transposon frequency (Figure 1) and to the incidence of a highly iterated palindrome (HIP1) sequence GCGATCC [41]. HIP1 sequences are greatly enriched in the genomes of most cyanobacteria except those of small marine *Synechococcus* and *Prochlorococcus* (Group 2 in Figure 1 and Elhai, unpublished results). Figure 3 shows that as the fraction of contaminating *Pmm* genes increased to 20%, the discriminating power of the W8 method dropped to zero, consistent with an earlier, more limited test [16]. Maximal discrimination by the GC% method was less affected, and maximal discrimination by codon bias was hardly affected at all. Contamination by *Tel* genes had a much lesser effect on all three methods.

**Figure 4:**
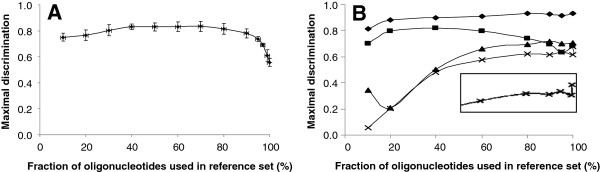
**Influence on maximal discrimination by choice of oligonucleotides used by W8 method.** The W8 method normally uses all octanucleotide frequencies in its reference set. Here, the method was modified so that only the n% octanucleotides with the lowest frequencies were used, where n varied from 10 to 100. In all cases, the target genome was contaminated to a level of 3% by foreign genes. (**A**) A HIP1-rich genome (that of *Syn*; 47.4% GC, 855 HIP1/million nt) was contaminated with genes from a HIP1-rich genome (from *Tel*; 53.9% GC, 1418 HIP1/million nt). The bars show standard deviations from repetitions with three different sets of contaminating genes. (**B**) A low-GC, HIP1-poor (36.4% GC, 2 HIP1/million nt) genome (*Pma*) was contaminated with genes from the HIP1-poor genomes of either *Pmt* (♦; 50.7 GC%, 39 HIP1/million nt) or *Cel* (■; 35.4 GC%, 5 HIP1/million nt). A high-GC, HIP1-poor genome (*Gvi*; ▲; 62.0% GC, 68 HIP1/million nt) or HIP1-rich genome (*Tel*; ×; 53.9% ?GC, 1418 HIP1/million nt) was contaminated with high-GC genes from *Syw* (59.4% GC, 64 HIP1/million nt). The inset shows at the same scale the spike near 100% usage of the reference set with *Tel* as the target genome.

Confining the range of oligonucleotide frequencies considered also had a striking effect on the results of the W8 method but not in the way we had anticipated. The W8 method was modified so that its set of reference octamers was taken from the lowest N percent of octameric oligonucleotides frequencies, with N varying from 10 to the usual 100. We tested 116 donor/recipient pairs, 20 in detail, and representative types of samples are shown in Figure 4. The manner in which maximal discrimination responded to varying the frequency slice was found to be quite sensitive to the presence or absence in the donor and recipient genomes of HIP1 sequences. When a HIP1-rich genome was used to contaminate another HIP1-rich genome, the maximal discrimination was little affected by the fraction of oligonucleotides used in the reference set, except for a large drop in discrimination when the 20% most overrepresented oligonucleotides were included (Figure 4A).

Less predictably, high-GC, HIP1-poor genomes contaminated with high-GC genes sometimes increased maximal discrimination as the frequency width increased, illustrated in Figure 4B (lower curves), while the maximal discrimination values of most other donor/recipient pairs were only modestly affected by the choice of frequency width (upper curves). Considering the overwhelming influence of HIP1 frequency on the efficacy of omitting high frequency oligonucleotides from the reference set (Figure 4B and Additional file 2 (Table 1); see also Discussion), we considered the possibility that the genomes without frequent HIP1 sequences might possess different high frequency sequences that influence the results. Table 1 shows how the varied results of suppressing high frequency nucleotides in the reference set becomes more organized if organisms are distinguished by their possession of frequencies of HIP1 sites and other octanucleotides. In brief, when the contaminating sequences are rich in HIP1 sites, a reduced reference set is better able to pick them out, but when they are rich in other octanucleotides and have a high GC%, a complete reference set is more effective. There is no strong preference of one method over another in other cases.

**Table 1:** **Change in maximal discrimination by W8 method in response to range of reference set**^***a***^

			**Source of contamination**^**b**^					
				**High GC**			**Low GC**	
			**High HIP1**	**High 8mer**	**Low 8mer**	**High HIP1**	**High 8mer**	**Low 8mer**
**Target**^**b**^	**High CG**	**High HIP1**	**+0.30 (2)**	***–0.47 (3)***	–0.03 (3)	**+0.15 (9)**	(0)	(0)
		**High 8mer**	–0.01 (3)	***–0.13 (6)***	–0.02 (3)	(0)	(0)	(0)
		**Low 8mer**	**+0.21 (4)**	***–0.05 (2)***	**+0.07 (3)**	+0.04 (8)	–0.02 (2)	–0.06 (0)
	**Low CG**	**High HIP1**	**+0.30 (8)**	***–0.07 (7)***	–0.04 (8)	**+0.12 (21)**	+0.04 (8)	–0.04 (2)
		**High 8mer**	(0)	(0)	–0.02 (1)	**+0.07 (10)**	+0.04 (3)	+0.09 (0)
		**Low 8mer**	(0)	(0)	(0)	(0)	(0)	(0)

^*a*^ The values shown are the compilation of results of 116 experiments in which genes taken at random from one genome were used to contaminate a target genome (see Methods for further explanation and Additional file 2 (Table 1) for a listing of the data on which the table is based). Each value shown is the difference of the maximal discrimination calculated using the W8 method subtracted from the value calculated using a modified W8 method in which the 40% least frequent octanucleotides were chosen as the reference set. Values in which the difference is at least 5 percentage points in favor of the 40% choice of octamers are rendered in bold. Those in which the difference is at least 5 percentage points in favor of the 100% choice of octamers are rendered in bold italics.

^*b*^ The categories of the source/target combinations were determined by whether the source and target genomes were high GC (> 50%) or low and by the frequency of the most frequent octameric sequence: **high HIP1** if the most frequent octamer is GCGATCGC (HIP1) and occurs at least 180 instances per million nucleotides, **high 8mer** if the most frequent octamer is not HIP1 and occurs at least 180 instances per million nucleotides, and otherwise **low 8mer**. The number in parentheses indicates the number of times the particular source/target combination was tested. Combinations were counted only if at least one condition (40% or 100% oligomers in reference set) yielded a maximal discrimination value between 0.10 and 0.90.

### Preliminary tests of parameters of the standard method

In order to address the problems of existing methods demonstrated in the previous section, we modified the W8 method in two ways: (1) by defining the reference set using core genes, highly conserved genes retained in almost all eubacteria and therefore unlikely to be of foreign origin (addressing the artifact illustrated in Figure 3), and (2) by limiting the oligonucleotides in the reference set to the most underrepresented 20% (addressing the common artifact illustrated in Figure 4A and Table 1). The value of 20% was chosen for historical reasons and is considered further in the Discussion section. The parameters of the method (henceforth called Core Gene Similarity, CGS) were tested as described below, by measuring the effect of changing these parameters on the maximal discrimination by CGS.

The CGS method relies on a set of genes, the core reference set, that one might expect to be relatively free of genes of foreign origin. However, if the set has just as high a frequency of foreign genes as those outside the set, then the method would not figure to have an advantage over the W8 method. To test the robustness of the method to contamination of the core reference set by genes of foreign origin, the set was intentionally contaminated by introducing genes that had the lowest CGS scores (furthest from core characteristics), as described later and provided in Additional file 3. Using the *Syn* genome as a test case, increasing contamination of the core reference set with putative foreign genes led to a drop in maximal discrimination, but the drop was slight when contamination was less than 20% (Additional file 4A). Contamination of the reference set had no effect in the case of *Pma* (Additional file 4B), a genome that appears to have few genes of foreign origin (see below). Contamination of the reference set actually improved maximal discrimination in the specific case where the genome with a relatively high GC fraction (*Pmt*) was supplemented with genes from high GC organisms (Additional file 4C and 2D), consistent with results described above, As one would expect, maximal discrimination per CGS was approximately equal to that per W8 when the reference set was contaminated by an amount comparable to the predicted level of foreign genes in the genome (see below and data not shown).

The CGS method appears therefore to be sufficiently robust to withstand low levels of foreign genes in the reference set. How high a level would one expect? We estimate that 6% of core genes have positions in phylogenetic trees discordant with a consensus organismal tree, based on an analysis of data presented by Zhaxybayeva et al. [42] on 1128 sets of orthologous genes from 11 cyanobacteria (see Methods for a more detailed calculation). The effective fraction may be significantly less, since many of the discordances indicated by the analysis of Zhaxybayeva et al. [42] are consistent with horizontal transfer events that took place so far in the past that the participating genes would not be recognized as foreign by CGS (see below). In brief, the occasional foreign gene in the core reference set would appear not to be a problem for the CGS method. The presence of foreign genes in the test-native set is another matter, one that is discussed in the next section.

One might imagine that scoring oligonucleotides from the coding strand would be more informative, as doing so might capture codon-specific tendencies. Alternatively, examining both strands might double the amount of information available. In fact, the choice made little difference in three of the four target genomes tested, while the coding strand was more effective in the case of one target organism (Additional file 5). We chose to use just the coding strand in subsequent calculations.

Tsirigos and Rigoutos [16] found that 8-nt was the optimal length in determining the set of reference oligonucleotides. We confirmed that 8-nt was generally more effective than 6-nt (Additional file 6). Our results (Additional file 7) were also consistent with those of Tsirigos and Rigoutos in their finding that covariance was the most effective statistical means of comparing oligonucleotide frequencies calculated from genes and the set of reference oligonucleotides. The number of genes in the set of core genes had only a minor effect on the ability to detect artificially seeded foreign genes (Additional file 8).

### Choice of threshold

Maximal discrimination will not be a useful measure for most who seek foreign genes in genomes. Instead, they will want a measure that can answer one of two distinct questions: which genes are most likely to be of foreign origin and which are most likely to have arisen by lineal descent. In either case, it would be helpful to have an estimate of error, i.e., the probability that a gene identified in one class is in fact a member of the other.

The descriptions of the W8 and codon bias methods provided empirical methods to calculate thresholds through which to predict foreign genes, and we wished to determine what fraction of false positives might be expected by using these thresholds. Tsirigos and Rigoutsos (see also Methods) placed the threshold at an inflection point in the distribution of scores obtained through the W8 method. When this procedure was applied to each of 24 cyanobacterial genomes not supplemented with test-foreign genes, 15% (SD = 5%) of test-native genes were identified as foreign (Additional file 9) and presumed to be false positives. Mrazek et al. [11] provided a complex formula that takes input from scores obtained from different classes of genes to arrive at a threshold. Using this threshold, the codon-bias method identified 7% (SD = 2%) of test-native genes as foreign (Additional file 9).

To permit a fair comparison of the methods under consideration, we chose to fix the thresholds for all methods such that each method produced a 5% putative false positive rate. It must be noted that if the test-native set contains genes of foreign origin with scores typical of foreign genes, then the threshold will be misplaced to the extent that false positives are replaced by foreign genes. For example, in the unlikely event that 10% of the test-native set are of foreign origin (unlikely -- see above), half of the foreign genes in the genome may have scores beyond the 5% threshold and thus go undetected.

### Efficacy of CGS method relative to other methods

The efficacies of the four methods considered in this work were compared with respect to their abilities to pick out test-foreign genes in a variety of genomes. However, since a natural genome is likely to pose quite a different challenge from one artificially seeded with foreign genes, we also tried to assess effectiveness of the methods using internal measures, the identification of transposases and genomic islands, and a comparison of predictions with phylogenetic trees.

**Figure 5:**
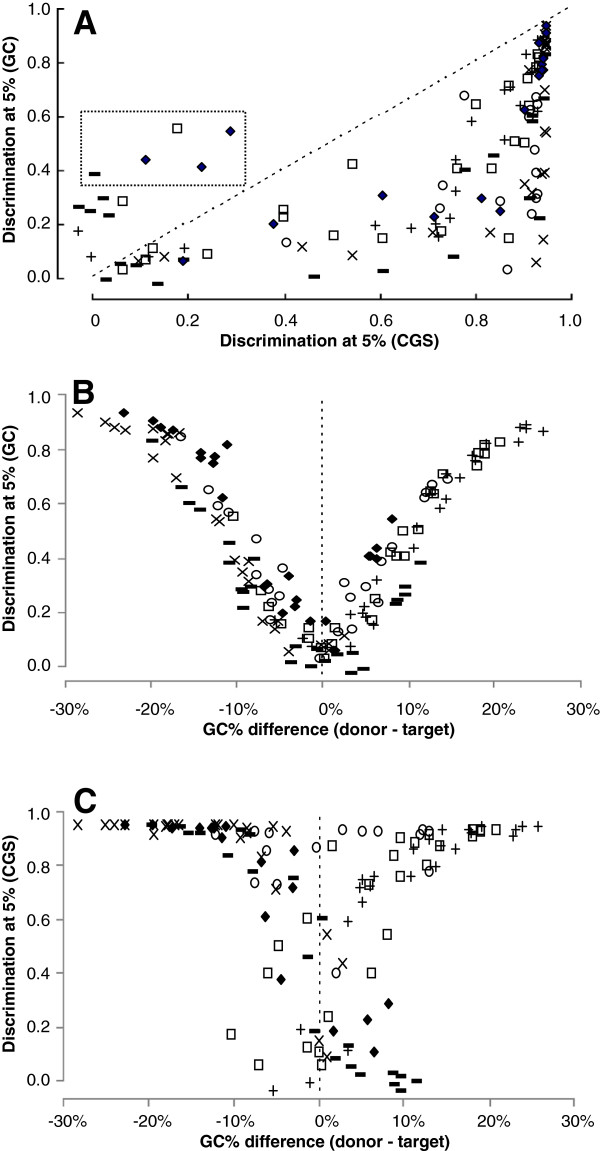
**Comparison of CGS and GC methods.** (**A**) Discrimination values based on CGS scores and GC scores were calculated using as targets the genomes of Group 1 cyanobacteria (□) *Ana*, (∆) *Mar*, and (○) *Syn*; of Group 2 cyanobacteria (+) *Pma*, (-) *Pmt*, and (×) *Syw*; and of (♦) *Tel*, contaminating them to a level of 3% with the same genes from up to 25 different organisms. Each point is the average of three trials. The values in the dotted box uses contaminating genes from (-) *Gvi* (GC% of 62%), (♦) *Gvi*, *Syw* (GC% of 59%), and *Cya* (GC% of 60%), (□) *Pmm* (GC% of 31%). (**B**) The same GC scores as in panel **A** are shown related to the difference in GC% of the donor and target genomes. (**C**) The same discrimination values as in panel **A** are shown related to the difference in GC% of the donor and target genomes. The identities of the genomes and values of both methods are provided in Additional file 2.

The CGS method was more effective than the GC method in 92% of the donor/recipient trials in picking out seeded foreign genes as judged by discrimination at a 5% false positive rate (Figure 5A), including all of the 127 cases where at least one of the two discrimination values exceeded 65%. The GC method did better in those cases where the GC content of the donor organism is extreme. One would expect that the GC method would suffer as the GC content of the donor and recipient approached each other, even when two genomes with similar GC content are evolutionarily distant, and indeed this was the case (Figure 5B).

Maximal discrimination was very poor (less than 0.4) when the two organisms differed by less than 5% in GC fraction. In contrast, the performance of the CGS method was much less tied to the GC contents (Figure 5C).

**Figure 6:**
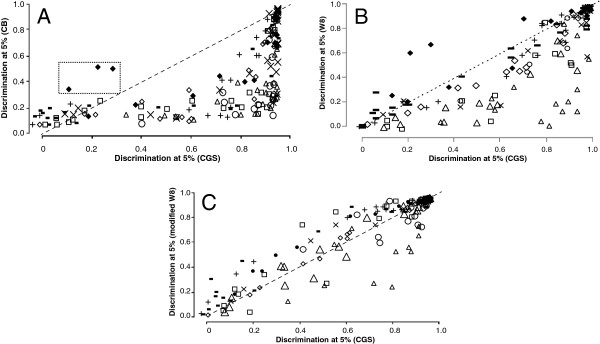
**Comparison of effectiveness of CGS method vs other methods.** Discrimination values determined by CGS, codon bias, W8, and modified W8 were calculated using as targets the genomes of Group 1 cyanobacteria (□) Ana, (∆) Cwat, () Mar, (○) Syn, and (◊) Ter; of Group 2 cyanobacteria (+) Pma, (-) Pmt, and (×) Syw; and of (♦) Tel, contaminating them to a level of 3% with the same genes from up to 25 different organisms. (**A**) Comparison with Codon Bias (CB). The values in the dotted box were obtained from cases in which contaminating genes were drawn from Gvi (GC% of 62%), Syw (GC% of 59%), and Cya (GC% of 60%). (**B**) Comparison with W8. The values in the dotted box were obtained from cases in which contaminating genes were drawn from from Gvi (GC% of 62%) and Syw (GC% of 59%). (**C**) Comparison with the W8 method modified to exclude contaminating genes. The W8 was modified so that genes artificially added to a genome did not contribute to the calculation of the reference set of octamer frequencies. The identities of the genomes and values for all methods are provided in Additional file 2.

The codon bias method fared no better in this test (Figure 6A), with CGS predicting seeded genes better than codon bias in all of the 97 cases where at least one of the two discrimination levels exceeded 65%. Codon bias appeared to be generally more effective in those trials where discrimination was poor, but this may be an artifact of the method. The apparent floor value of 0.10 to 0.25 for codon bias is consistent with a failure in our assumption that the set of test genes (which excludes core genes) is a random sampling of all native genes (including core genes) with respect to the measured quantity. Indeed, confining the analysis to 80% of the genes of *Syn* that have the most normal scores, the average codon bias score for test-native genes is far from the average score for all genes in the set and close to the average score for the highly expressed genes of the training set. In contrast, the average CGS score for test-native genes is indistinguishable from the average score for all genes of the set (data not shown). Codon bias would therefore identify more genes as foreign but at the cost of also identifying more native genes as foreign (see below).

The W8 method was more effective in this test than the previous two methods (Figure 6B), as CGS had a higher rate of success than W8 in only 76% of the cases. However, CGS was more successful in almost all the cases where the difference was major.

The W8 and CGS methods are very similar, differing only in the reference frequency set (all octamers for W8 vs least frequent octamers for CGS) and the choice of reference gene set (all genes for W8 vs core genes for CGS). In formulating the CGS method, we confined the reference set to core genes thinking that doing so would avoid the poisonous influence of foreign genes in the genome. To test how great this influence might be, we modified the standard test of W8 so that artificially seeded genes were tested one at a time, without affecting the set of reference frequencies (calculated with no seeded genes), and we calculated the frequency set using the same infrequent octamers as used for CGS. With these modifications, the only difference remaining between the two methods was that the modified W8 method used all genes as the basis of the frequency set, while CGS used only core genes. If pre-existing foreign genes affected the frequency set, then it should be apparent by a comparison between CGS and modified W8. Of course it is not possible to exclude from the W8 calculation foreign genes in the genome, except in artificial tests such as these.

The results of such a comparison are shown in Figure 6C. In accordance with results presented in the last section, the discrimination ability of the modified W8 method was much improved relative to the unmodified W8 method. However there still remained a difference in discrimination relative to CGS. The modified method was more effective, particularly with the genomes of the marine *Prochlorococcus* and *Synechococcus*, but less effective with the genomes of those organisms with highest density of transposons.

**Figure 7:**
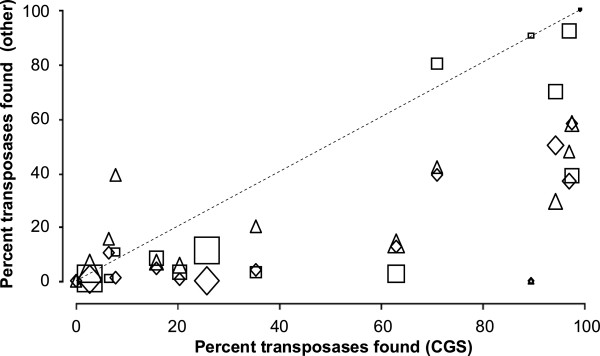
**Detection of transposases by different methods compared to CGS.** Transposases from the 15 cyanobacterial genomes considered in this study with annotated transposases were predicted to be of foreign origin if their scores went beyond the threshold that excluded all but 5% of the test-native set. The fraction of transposases found for a given organism by the CGS method was compared to the same fraction found by the W8 (□), codon bias (∆), and GC (◊) methods. The area of the symbol is proportional to the fraction of the genome attributable to transposases (Additional file 9).

One way to assess the effectiveness of the different methods without the artificiality of seeding a genome with exogenous genes is to identify genes that probably arose by lateral transfer and examine the success the methods in identifying them. Transposases are plausible candidates [43], though it must be borne in mind that they, like viruses, can establish a close relationship to a group of related organisms. Figure 7 shows the success the W8 and codon bias methods had relative to CGS in detecting transposases in 15 cyanobacterial genomes where transposases have been annotated. It is not surprising that W8 suffers by comparison, since repeated sequences such as transposons contribute to the reference set roughly in proportion to their numbers. Codon bias also did not do well, comparable to its showing with seeded foreign genes (Figure 6A).

Another source of presumed foreign genes is the set of genes in genome islands. Coleman et al. [44] compared the genomes of *Pmm* and *Pmi* and identified genome islands in each strain, presumably having arisen by lateral transfer. We used the 210 coding genes from *Pmm* and the 246 coding genes from *Pmi* lying within genome islands in tests of the methods (Table 2). No method detected more than 30% of the genes in the islands. Codon bias picked out the most but also identified far more genes as putative foreign than the other methods. As judged by a percentage of putative foreign genes confirmed by localization in genome islands, CGS was as good as codon bias or better. W8 did not find genes within the islands at a rate distinguishable from chance.

**Table 2:** Putative foreign genes in genome islands

		**Island hits**^**c**^		
**Method**^**a**^	**Total putative foreign**^**b**^	**Expected**	**Observed**	**Observed/Total**^**d**^
*Pmm* (*Prochlorococcus marinus* Med4)^e^				
Codon bias	231	28	60^*^	0.260
CGS	91	11	23^*^	0.253
W8	96	12	15	0.156
GC	109	13	13	0.119
*Pmi(Prochlorococcus marinus* MIT9312)^e^				
Codon bias	272	37	56^*^	0.206
CGS	118	16	29*	0.246
W8	107	15	16	0.150
GC	119	16	25^*^	0.21

^a^ All methods used a threshold that excludes 95% of the test-native set.

^b^ Genes identified as foreign by the method in the given genome. *Pmm* has 209 coding genes in genome islands out of a total of 1713 coding genes, and *Pmi* has 246 coding genes in genome islands out of a total of 1810 coding genes, per Coleman et al. (2006). ^c^ The number of genes identified as foreign by the method that lie within a genome island. The expected number is that which would arise if the genes identified as foreign were distributed randomly throughout the genome. Asterisks indicate statistical significance to the level P < 0.001. Numbers without asterisks are not significant even to P < 0.05. ^d^ The fraction of total putative foreign genes identified by the method that lie within genome islands.

Perhaps the ideal test is a comparison of the predictions of each method against discordances found from analyses of phylogenetic trees. Zhaxybayeva et al. [42] presented 131 trees, based on sets of well conserved orthologous genes, that had a total of 135 conflicts with respect to an organismal tree, each associated with a discordant pair of genes. Surprisingly, in only 9% of these reported conflicts was a gene predicted by CGS to be of foreign origin included in the discordant pair. The other methods did no better (data not shown). While this fraction is significantly greater than that predicted by chance (p < 1% per *χ*^2^), it nonetheless seemed low, so we examined the trees in detail. All 12 conflicts from Zhaxybayeva et al's list that name a gene with a low (< 0.05) CGS score were examined, along with 9 others chosen at random (2 involving the same gene) with higher CGS scores, in order to assess why the reported conflict was not detected by the CGS method. All the trees are provided in Additional file 10. They differ from those of Zhaxybaeva et al. in that they consider sequences from up to 26 cyanobacteria (instead of 11) and use a Bayesian approach and maximum likelihood to construct trees, as described in Methods, instead of quartet analysis. Trees from the two studies are often inconsistent, and in some cases the additional sequences in our trees reveal paralogous relationships that resolve the reported conflicts.

**Figure 8:**
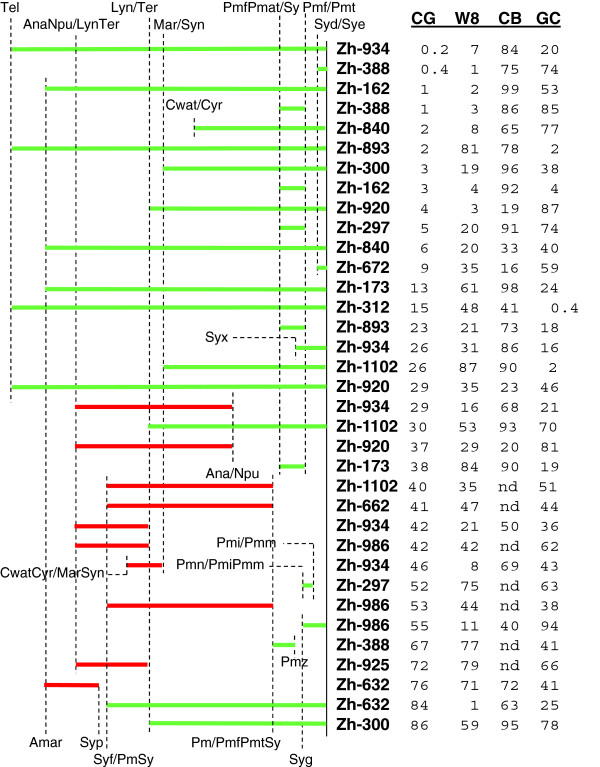
**Predicted time ranges of horizontal transfer events.** Evolutionary time ranges symbolized by horizontal lines are shown during which horizontal gene transfer events may have occurred to explain the phylogenetic trees provided in Additional file 10. Each line is associated with a set of proteins reported by Zhaxybayeva et al. [42] to contain at least one conflict with the 16 S rRNA gene tree. The termini of the time ranges are defined by evolutionary events deduced from the 16 S rRNA gene tree (Figure 1), either the divergence of a single organism (represented by the symbol of that organism) from many or the divergence of one or two organisms from one or two other organisms (represented by the diverging organisms symbols separated by a slash). Evolutionary time proceeds left to right, roughly proportional to the number of mutations that have accumulated in ribosomal DNA. The scores of the gene or genes (averaged) predicted to have resulted from horizontal gene transfer are given at the right, according to the four methods considered. Green lines indicate time periods that are at least partially as recent as the divergence of *Pmf*/*Pmt* from *Synechococcus.*

The trees were interpreted, extracting from each a time range of the simplest horizontal gene transfer events that could have produced well supported discrepancies with respect to the 16 S rRNA gene tree (Figure 1). In many cases, multiple events are required. A summary of the analysis is shown in Figure 8. The time ranges of all 10 of the events predicted by CGS are consistent with HGT events that have occurred more recently than the divergence of Pmf and Pmt from the remaining marine cyanobacteria. Although many potentially recent HGT events thus defined were not predicted by CGS, the probability is less than 5% per *χ*^2^ analysis that 10 potentially recent HGT events would arise from a random sampling of all the events considered. Four *syn* genes chosen at random from those with CGS scores less than 0.05 were also analyzed. In each case, their trees showed strong evidence of HGT (Additional file 10).

### Survey of putative foreign genes in cyanobacteria

One might reasonably expect that different genomes would carry different fractions of genes of foreign origin. We applied three of the methods to the genomes of 25 cyanobacteria, asking how many genes fell beyond a threshold score determined by a 5% false-positive rate. Codon bias almost always identified more putative foreign genes for a given genome (consistent with the failure mentioned earlier of the assumption that the 5% threshold determined from test-native genes corresponds to to the level appropriate to identify 5% of all native genes), and the GC and W8 methods generally identified fewer putative foreign genes relative to CGS (Additional files 9 and 11). A possible reason for this was discussed in the previous section. For now we will focus on the values reported by CGS, which are given for all genes in Additional file 3.

**Figure 9:**
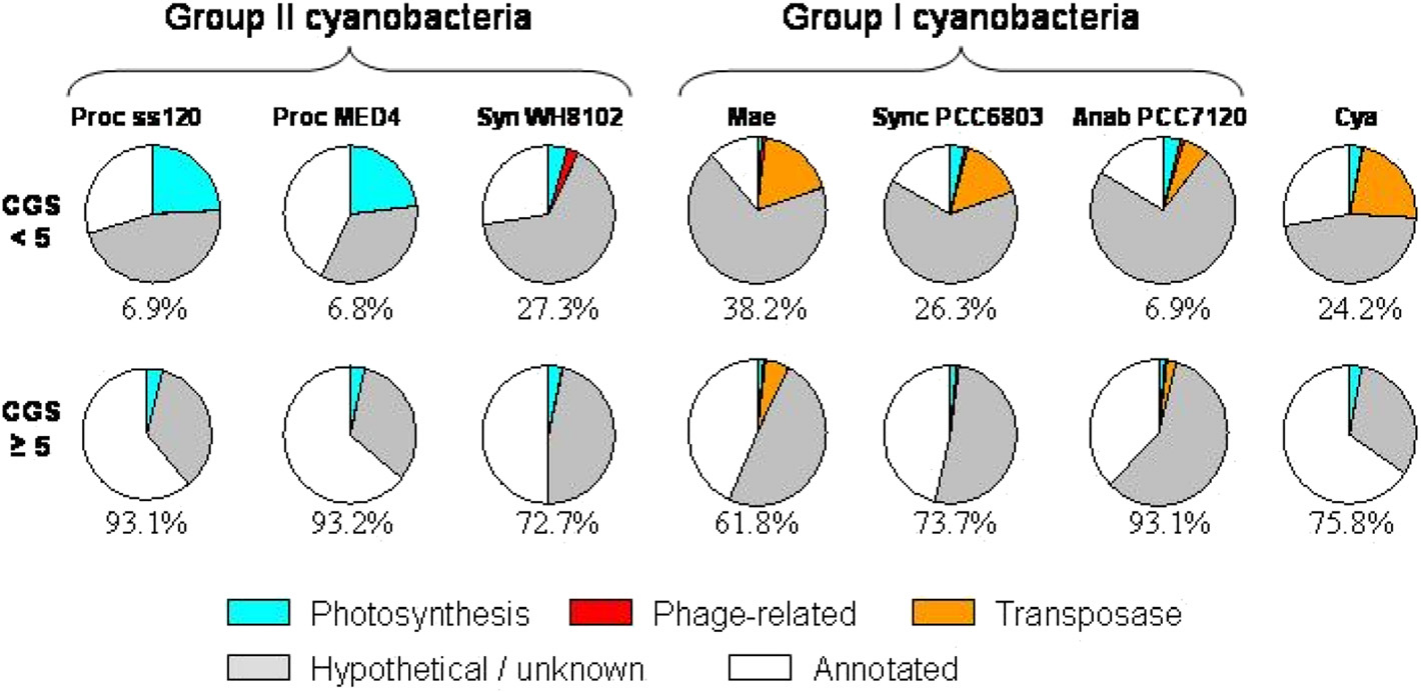
**Function of genes identified as putative foreign.** The distribution of genes in seven representative cyanobacteria is shown, in each case dividing the genes into two classes: those with CGS scores < 5 (top row) and those with scores ≥ 5 (bottom row). The circle represents all genes of the class.

The number of foreign genes identified was correlated with the clade of the organism (Figure 9 and Additional file 9). Reasonably enough, the fewest foreign genes were found in genomes of the low-GC *Prochlorococcus* of Group 2, which are the smallest genomes amongst cyanobacteria, ranging from 1.66 to 1.84 MB for the five genomes in this category. Four of the five have fewer than 8.9% putative foreign genes per CGS, which should be interpreted as 3.9%, as the chosen threshold should lead to a misidentification of 5% of native genes. The fifth, *Pmz*, is also the only *Prochlorococcus* with identified transposases (five).

The four genomes from the filamentous cyanobacteria also have a low reported incidence of putative foreign genes per CGS, all less than 8.4% (calculated 3.4%), despite a range of genome sizes from 7.04 to 9.06 MB.

The middle and high-GC marine *Prochlorococcus/Synechococcus* of Group 2 have larger genomes (2.23 to 2.68 MB) and a relatively high fraction of putative foreign genes (17.5% to 32.5%, calculated 12.5% to 27.5%). The unicellular members of Group 1 have the broadest taxonomic range and the broadest range of putative foreign genes, from 11.6% (*Cyanothece* PCC 8801) to 38.2% (*Microcystis aeruginosa* NIES 843).

As might be expected, the set of putative foreign genes is heavily biased towards transposons and phage sequences when such are present in the organism, and the same is true for genes that have no known function (Figure 9). Surprisingly, genes related to photosynthetic energy production are also overrepresented in the set of putative foreign genes, at least in the case of Group II cyanobacteria. Genes that are highly expressed, including photosynthesis genes, have unusual codon preferences (11), which could conceivably affect CGS scores, but this phenomenon is unlikely to account for the observed bias, as we were unable to detect any obvious correlation between gene expression and CGS score in *Ana* and *Syn*, two organisms for which microarray data is available (data not shown).

**Figure 10:**
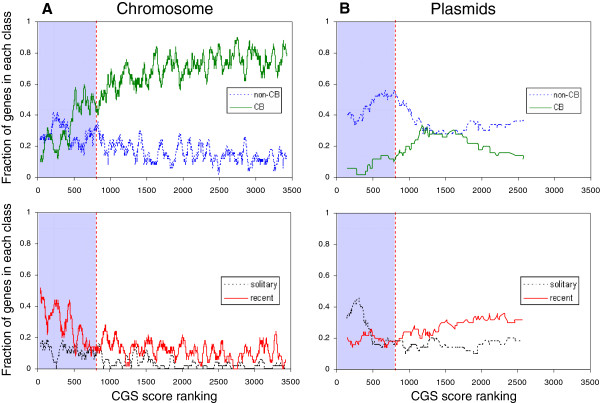
**Evolutionary context of genes as related to CGS scores.** The proteins encoded by the chromosome (**A**) or five plasmids (**B**) of *Synechocystis* PCC 6803 were ordered by their CGS scores and divided into four categories: cyanobacterial, non-cyanobacterial, recent origin, and solitary, as described in the text and Methods. Each point on the graph is a frequency based on 50 proteins centered around the CGS ranking. The blue shaded areas indicate genes with CGS scores < 0.05.

Foreign proteins would also be expected in general to show greater similarity to proteins of distantly related organisms than would native proteins. To assess whether the proteins identified by CGS scores had this property, we counted the numbers and sources of similar proteins according to Blast, a crude measure but one that could be practically applied to all proteins in an organism. Figure 10 shows how the number of proteins in *syn* vary according to their CGS scores when classified by their evolutionary context. In brief, the context of a protein was termed *cyanobacterial* when matches were primarily to cyanobacterial proteins, *recent* when there were few matches but they were to proteins of closely related cyanobacteria, *non-cyanobacterial* when matches were primarily to proteins from outside the cyanobacteria, and *solitary* when the only match was to the protein itself (see Methods for precise definitions).

Chromosomal *Syn* proteins that gave low CGS scores were much less likely than those with high CGS scores to have a cyanobacterial context (Figure 10A). Furthermore, the low CGS proteins were much more likely to be of apparently recent origin. Plasmids are generally transient components of a genome, and this is reflected in the greater fraction of their proteins with non-cyanobacterial contexts and those that are solitary (Figure 10B and Additional file 12). This is especially evident in proteins with CGS scores < 0.05, where 72% of the proteins are in these two categories.

## Discussion

There is great appeal to the idea of identifying genes of foreign origin as easily as one identifies the genes themselves, through information obtained directly from the genome, possibly supplemented by readily available outside resources. The analysis of compositional features of genes offers that prospect, but the operational simplicity comes at a cost: a layer of abstraction between the measure and the phenomenon. Very little is understood as to why compositional features are conserved amongst like organisms, and so caution must be exercised in employing such features as surrogate measures of horizontal genetic transfer. We have attempted to identify parameters of oligomer frequency analysis important in identifying genes of foreign origin and propose a method that may often perform better than existing surrogate methods.

The proposed CGS method performed better than GC fraction, codon bias, or W8 in the great majority of trials artificially introducing foreign genes into genomes (Figures 5 and 6), overwhelmingly so when discrimination between foreign and core genes was high (i.e. when one method or the other could distinguish with confidence foreign from native genes). The tests attempted to simulate the acquisition of foreign genes in the recent past, before sufficient time has elapsed for the genome-specific composition features to fade [12]. One might argue that these seeding experiments are too unnatural to be fully convincing. We therefore sought tests of the methods using extant genes that are plausibly of foreign origin. The CGS method was considerably more effective than any of the other methods tested in flagging resident transposase genes as foreign (Figure 7). It was also clearly superior to either W8 or GC in finding genes within genome islands (Table 1).

What is the basis of the superior performance of the Core Gene Similarity method? As the name suggests, a distinctive feature of the method is its reliance on well conserved core genes, to define the training set without contamination by foreign genes that may be present in the genome. Calculating the training set using all genes rather than core genes drastically degraded performance when the genome was artificially seeded with foreign genes at a level of 20% (Figure 3), a reasonable value for natural genomes ( [2] and Additional file 9). Conversely, the conceptual removal of foreign genes from the training set greatly improved the performance of the W8 method (compare Figure 6B with Figure 6C).

A second distinctive feature of the method is that it limits the training set of oligomers. We initially considered that confining the training set to underrepresented octamers would increase the method’s ability to detect genes of foreign origin. This second hypothesis turned out not to be true, since for most simulations, there was little difference in discrimination when using only underrepresented octamers as compared to including the middle range of octamers (Figure 4). On the other hand, the inclusion of overrepresented octamers caused in most simulations a sharp drop in performance (Figure 4 and Table 1), correlated with the presence of high frequency HIP1 sites in contaminating genomes. Two of the most striking observations shown in Table 1 are readily explained. First, if high frequency HIP1 sites are retained in the training set, along with high frequency sequences one nucleotide removed from HIP1 (J. Elhai, unpublished results), then their presence in foreign genes would present a strong but misleading signal of the origin of that gene. Second, ignoring high frequency oligomers reduces the ability of the method to discern foreign genes, when those genes come from high GC organisms with high frequency oligomers differing from those in the target genome (Table 1). Empirically, the optimal general solution seems to be to construct a training set from the 80% least frequent octamers, a practice that retains the benefit described in the first case and excludes almost all the injury described in the second case. Understanding why this is would require insight into the nature and spectrum of repeated sequences in bacterial genomes, a goal we are pursuing.

Some of the limitations of compositional methods to detect horizontal transfer are well known. The sequence signature of the originating genome must lessen with time [12]. Events occurring in the distant past may be invisible to compositional methods, and Figure 8 provides evidence for this, just as recent events may prove difficult for phylogenetic methods to detect [45]. Compositional methods may also founder because of failure of the basic assumption that the measured feature is constant over the genome, except at locations of horizontal transfer. It has been reported that G + C in the third codon position varies along the length of the *E. coli* genome [46], and strand-specific deviations from randomness in the location of oligomers are well known [47]. Combinations of methods may be more effective in detecting instances of horizontal transfer [7]. The current work makes clear that changing the parameters of even a single method may be important in optimizing the detection of certain combinations of source and target genomes.

It must also be noted that the method described here is not capable of detecting horizontal transfer of genes in the reference set. However, this restriction is easily sidestepped by running the algorithm twice with nonoverlapping sets, at the minor cost of doubling the execution time.

The most significant limitation of this work is that it focuses on a single class of organisms, the cyanobacteria, most of which are known to possess high frequency HIP1 sites (Figure 1). While very few bacterial genome possess octomers as frequent as HIP1 sites (J. Elhai, unpublished), many have other significantly repeated sequences, such as recombinogenic chi sites [48] and transformation uptake signals [49], which may also affect the performance of surrogate methods in a similar fashion as HIP1 sites.

## Conclusions

CGS scores provide a means by which thousands of genes can be evaluated for horizontal gene transfer in a few minutes of computer execution time. The use of the test-native gene set as an internal control enables the user to adjust parameters in a rational fashion to allow greater sensitivity at the expense of accuracy or vice versa. It is important to note that the method does not require any prior knowledge of species related to the target organism(s), as the reference and test-native sets are derived using genes that are common to all eubacteria.

## Methods

### Genomes and general analytical methods

The genomes used in this study and their sources are listed in Figure 1. Over 3400 computational experiments and counting of octamers were performed within the integrated knowledge/programming environment of the CyanoBIKE instance of BioBIKE [26]. The numeric results of those experiments described in this article are given in Additional file 2, and the code is available on request. HIP1 sites were counted using BioBIKE's COUNT-OF function.

### Phylogenetic trees

Phylogenetic trees were inferred using a Bayesian approach or maximum likelihood, as indicated, based on alignments obtained using MAFFT [50] (in the of case 16 S rRNA gene trees) or with guidance from the corresponding protein sequences, using PAL2NAL [51] (in the case of other gene trees) provided with protein sequences aligned with MAFFT [51], using the E-INSI method and default parameters. Positions that can be reliably used in phylogenetic analysis were extracted with Gblocks [52].

Bayesian trees were constructed using BEAST 1.6.1 [53], based on a GTR + I + G model with 4 categories of substitution rate. The Bayesian Monte Carlo Markov chain reconstruction was run for 50 millions generations and trees were sampled every 1000 steps, with the first 10 thousand trees discarded. The maximum clade credibility tree was obtained with TreeAnnotator 1.6.1. Bayesian posterior probabilities greater than 0.5 are indicated at the nodes (0.8 in the case of the 16 S rRNA gene tree).

Maximum likelihood trees were constructed using PHYML [54] based on a GTR + I + G model using 4 categories of substitution rate and a Gamma distribution parameter estimated by PHYML from the data set. The GTR + I + G model determined to be the most appropriate to our data set according to the Perl script MrAIC1.4.3 [55].

The evolutionary distance between two sequences was obtained by adding the lengths of the horizontal branches connecting them, where the full horizontal length shown is 0.1 mutation per position.

### Core gene similarity (CGS) method

#### Overview of steps

To predict genes that came to the genome under consideration (the *target genome*) by horizontal gene transfer (*putative foreign genes*), a set of *reference genes* within the genome was selected, consisting of *core genes* with orthologs in a set fraction of eubacterial genomes. From these, a set of *reference oligonucleotides* was calculated using the least frequent oligomers in the genome. A second set of genes, the *test-native* genes, was determined as those with orthologs in a representative set of *reference organisms*. The frequencies of these reference oligonucleotides were calculated in all protein-encoding genes of the genome and compared with the frequencies of the core genes, to produce a *value of merit*. A threshold value was determined as that which all but 5% test-native genes were excluded. Genes that have values beyond this threshold were predicted to be of foreign origin. The method was evaluated in part by seeding the genome with a set of *test-foreign* genes taken from different genomes. Each of these steps is described below in more detail.

#### Determination of the set of core genes

Genes from *Syn* with orthologs in the genomes of 13 diverse cyanobacteria (see below) were used to scan for orthologs in the 717 eubacterial genomes in KEGG [56] as of October 20, 2008. Orthologs were defined using the KEGG best-best option (bidirectional best hit), with a Smith-Waterman scores of at least 100. The greatest number of orthologs for a given gene was 702. In the standard method, those *Syn* genes that found orthologs in more than 90% of this maximum (i.e., in more than 631 eubacterial genomes) were used to find orthologs in the target genome. These orthologs were collected as the core genes. Typically, a cyanobacterium had about 217 core genes determined in this way.

#### Determination of the sets of reference oligonucleotides and reference frequencies

The frequency of each octamer in the core genes was determined as the sum of the counts of the 8-mer in each core gene divided by the sum of their effective lengths (the length of each gene minus 7). The octamer-frequency pair was sorted by frequency and the 20% with the lowest frequencies were collected and called the reference oligonucleotides. In many cases, more than 20% of the octamers produced no counts. In such cases, the reference oligonucleotides included all octamers that produced no counts.

#### Determination of the set of test-native genes

The set of test-native genes could have been obtained in the same manner as described above for set of core genes, simply by reserving part of the core genes for testing. However, we actually obtained the set in the way described below, made trivial by the built-in capabilities of BioBIKE. In fact, the two sets substantially overlap.

A set of genes in the target genome with orthologs in all of a representative subset of cyanobacteria (*Ana Ava Cwat Gvi Npu Pma Pmm Pmt Sef Syn Syw Tel Ter*) was determined, using the COMMON-ORTHOLOGS-OF function of BioBIKE. Orthologs were defined as bidirectional best hits per Blast [57], with E-values better than 10^-10^. Those genes in this set were called the test-native set. Genes in the core gene set were excluded from the test-native set so as not to give an unfair advantage to the CGS method, which uses the core gene set to calculate the reference frequencies.

#### Determination of the set of test-foreign genes

All protein-encoding genes from an organism distinct from the target organism were placed in a random order and saved in a file. For a computational experiment, a specified number of genes were taken from the file and called the test-foreign set. In this way, each experiment using a given number of genes used the same genes. The number was determined as a given fraction of the final artificially seeded genome. For example, the number of foreign genes to seed a genome to a level of 3% was determined as F in the equation F/(F + N) = 3%, where N is the number of protein-encoding genes in the target genome.

#### Calculation of value of merit

The frequency of each oligonucleotide in the oligonucleotide reference set was calculated for each protein-encoding gene of the target organism (and in some tests of the method, for each gene in the set of test-foreign genes). These frequencies were compared to the set of reference frequencies by means of a covariance test:

(None) $$\text{raw}-{\text{CGS}}_{g}=\frac{1}{n}\overset{n}{\underset{k=1}{\sum }}f_{g}*f_{R}$$

Where *f*_*g*_ and *f*_*R*_ are the frequencies for a given category within the gene and the core genes, respectively, and the sum is taken over *n* categories. The categories consisted of each oligonucleotide of the set of reference oligonucleotides plus an extra category consisting of all other oligonucleotides combined. Taken in this way, raw-CGS_g_ is higher for genes whose frequencies are similar to those of the reference set and lower for genes whose frequencies are dissimilar. The final CGS_g_ score of a gene is calculated by determining the fraction of test-native genes with raw-CGS_g_ scores less than the raw-CGS_g_ score of the gene.

#### Determination of threshold CGS value

The *5% threshold* was determined by finding a score that divides the values of merit of the set of test-native genes into two groups: 5% below the threshold and 95% above. In many tests, a different threshold was determined, one that maximized the difference between presumed true positives (test-foreigns scoring below the threshold) and false positives (test-native genes scoring below the threshold). This was done by sorting the calculated values of merit and testing each as a possible threshold until the conditions were met.

### Existing methods

To implement the GC method, the counts of G + C nucleotides in each gene was compared by means of a *χ*^2^ test to the counts expected in the gene based on the frequency of G + C over all protein-encoding genes. This procedure differs from that used by Lawrence and Ochman [12] and others, measuring differences in G + C in the third position of codons.

The Codon Bias (CB) method was implemented essentially as described by Mrázek et al. [11]. The lists of reference genes (translation processing factors, chaperones, and ribosomal proteins) were obtained for each genome by searching its gene annotations for relevant terms, using BioBIKE's GENES-DESCRIBED-BY function. The parameter *M* used by Mrázek et al was considered to be adjustable. To achieve discrimination at a level of 5%, *M* was set so that 5% of test-native genes were predicted to be foreign (or PA in the language of Mrázek et al.). This procedure differs from the Codon Adaptive Index [58] used by some.

The W8 method was implemented according to Tsirigos and Rigoutsos [16]. Covariance was calculated as described above for CGS. In most tests, the set of reference frequencies was calculated using all octamers, as described by Tsirigos and Rigoutsos, but in some tests a subset of octamers were used, determined as described above. When foreign genes were artificially seeded in the genome, the octamer frequencies were calculated over all genes in the target genome, including the foreign genes. In some tests the method was modified so that the frequencies were calculated excluding the foreign genes.

For most experiments, the threshold was determined as described above for the CGS method. In cases noted in the text, however, the threshold was determined essentially as described by Tsirigos and Rigoutsos. Specifically, we smoothed the curve of W8 scores (sorted by value) by averaging over a moving window of 100 points. Then the derivative calculated at each point was compared to the derivative averaged over the central 80% of the curve (the constant region). The lowest value with a derivative greater than the average derivative was defined as the threshold.

### Calculation of expected foreign genes in the reference set

Zhaxybayeva et al [42] examined 1128 sets of orthologous genes from 11 cyanobacteria and found that 443 (39%) had no conflict with the consensus organismal tree. Tree analyses for 131 of the remaining gene sets were reported, and within them, 135 pairs of genes were connected in a way discordant with the consensus organismal tree (127 of the gene sets exhibited a single discordance and 4 exhibited two). Since the 131 gene sets contained 1355 genes (9 to 11 genes per set), 10% of the genes in these gene sets are apparently discordant. If these gene sets are representative of all the gene sets with discordances, then overall, 6% (10% * 685/1128) of genes in the orthologous sets show evidence of horizontal transfer.

### Evolutionary context

With the goal of assessing whether proteins are most similar obtaining a crude assessment of the number of proteins All matches had E-values greater than 0.001, and only those matches were considered that were better than the last best match to any member of Group I cyanobacteria (see Figure 1).

## Competing interests

The authors declare that they have no competing interests.

## Authors’ contributions

JE conceived the experiments, HL and JE executed the experiments, AT performed phylogenetic analysis and created tools necessary for identifying core genomes, HL drafted the manuscript, JE wrote the manuscript. All authors read and approved the final manuscript.

## Supplementary Material

Additional file 1 **Support for phylogenetic tree inferred from 16 S rRNA gene sequences **[26,59-64]. Click here for file

Additional file 2 Experimental results summarized in this work. Click here for file

Additional file 3 CGS scores of genes. Click here for file

Additional file 4 Genomes with contaminated cores, seeded with genes from other genomes. Click here for file

Additional file 5 Comparison of CGS method using one strand or both strands of genome. Click here for file

Additional file 6 Comparison of CGS method using hexamer or octamer frequencies. Click here for file

Additional file 7 Comparison of covariance and G-score methods. Click here for file

Additional file 8 Influence of size of reference set on maximal discrimination. Click here for file

Additional file 9 Putative foreign gene summary in genomes without artificial contamination by foreign genes. Click here for file

Additional file 10 Phylogenetic trees of conserved genes with reported conflicts. Click here for file

Additional file 11 Fraction of genes identified as putative foreign by different methods compared to CGS. Click here for file

Additional file 12 **Evolutionary context of genes of *Synechocystis* PCC 6803.** Click here for file
