# Supplementary material for: Detection of horizontal transfer of individual genes by anomalous oligomer frequencies
Source: BMC Genomics. 2012 Jun 15;13:245. doi: 10.1186/1471-2164-13-245 (PMC3497702; doi:10.1186/1471-2164-13-245)
Supplement: Additional file 1 — Support for phylogenetic tree inferred from 16 S rRNA gene sequences [26,59-64]. [file 1471-2164-13-245-S1.pdf]

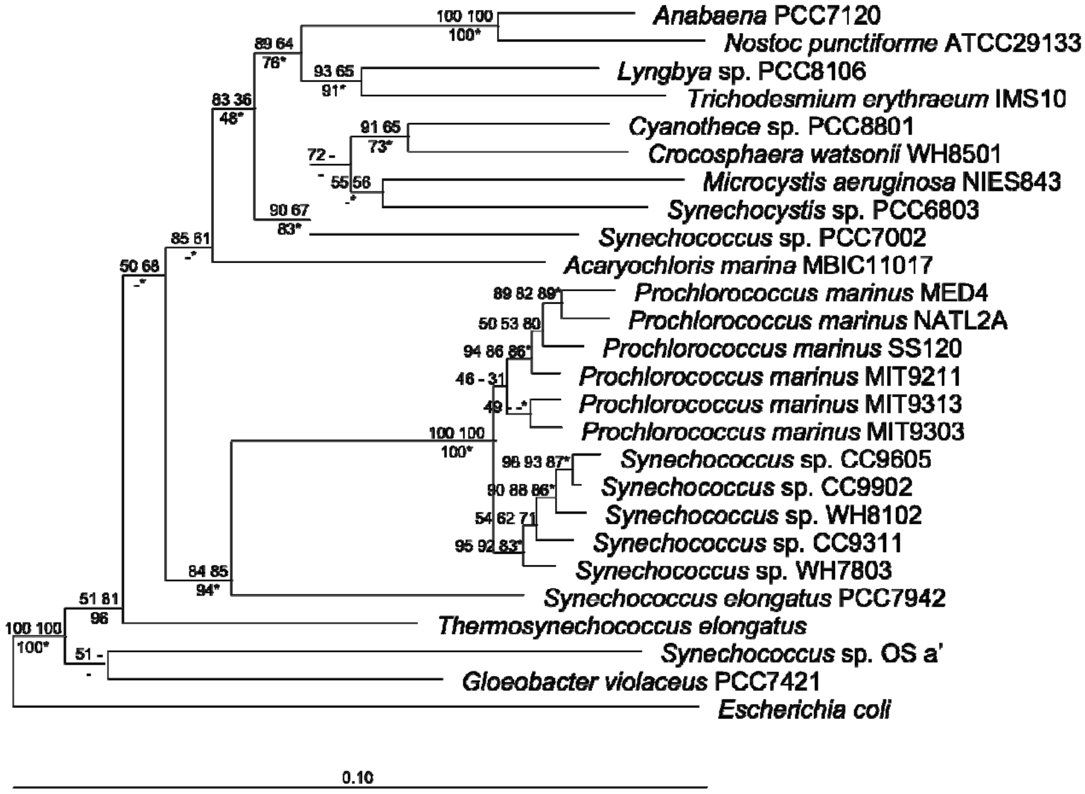

Additional File 1: Support for phylogenetic tree inferred from 16S rRNA gene sequences. The phylogenetic tree shown was inferred from 16S rRNA gene sequences by neighbor joining using the Jukes and Cantor model for multiple nucleic acid substitutions. The estimated evolutionary distance between two sequences was represented by adding the lengths of the horizontal branches connecting them and using the scale bars (0.1 mutation per position). Support for this tree was assessed by four methods: (1) the neighbor joining method on a Jukes and Cantor distance matrix implemented in ARB [59] (indels and ambiguous nucleotides were not taken into account in the distance matrix calculation); (2) the Wagner parsimony of DNAPARS [60] implemented in ARB; (3) the maximum likelihood of PHYML [40] based on a GTR+I+G model using 4 categories of substitution rate and a Gamma distribution parameter estimated by PHYML from the dataset, with the GTR+I+G model determined to be the most appropriate to our data-set according to the Perl script MrAIC1.4.3 [61]; (4) the maximum likelihood of TREE-PUZZLE 5.2 [62] using the quartet puzzling algorithm [63] to reconstruct the tree topologies based on 50000 puzzling steps and the GTR model of substitution [64], with substitution rates determined beforehand by PHYML, using 4 categories of substitution rate and the Gamma distribution parameter estimated by TREEPUZZLE from the data-set. Bootstrap values obtained using neighbor-joining (1000 trees), parsimony (1000 trees) and maximum likelihood (100 trees) are indicated at the nodes when equal to or greater than 50%. An asterisk indicates nodes with a treepuzzle (50000 puzzling steps) support value equal to or greater than 50. Comparisons of conserved nodes between the four tree construction methods were facilitated by the software package ARB and specific scripts written with the Biological Integrated Knowledge and Programming Environment BioBIKE [26].
