# Supplementary material for: Detection of horizontal transfer of individual genes by anomalous oligomer frequencies
Source: BMC Genomics. 2012 Jun 15;13:245. doi: 10.1186/1471-2164-13-245 (PMC3497702; doi:10.1186/1471-2164-13-245)
Supplement: Additional file 4 — Genomes with contaminated cores, seeded with genes from other genomes. [file 1471-2164-13-245-S4.pdf]

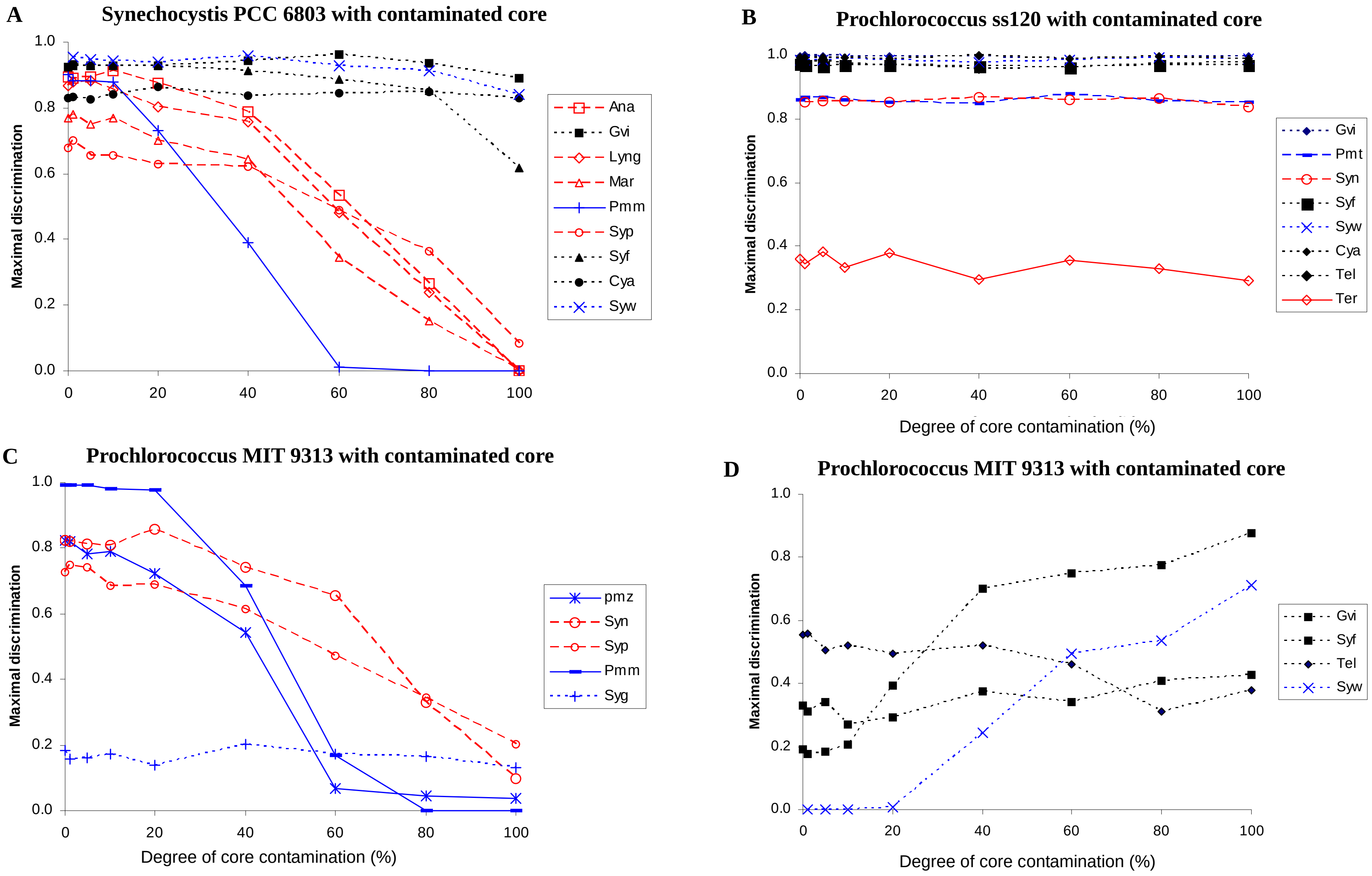

Additional File 2: Genomes with contaminated cores, seeded with genes from other genomes. The indicated percentage of the target organism's set of core reference genes was replaced by genes taken at random from the set of genes from the same organism with the lowest CGS scores. Then the genome was supplemented to a level of 3% by genes from the other indicated genome. Group 1 and Group 2 organisms are represented by lines and symbols that are red and blue, respectively. Low GC% genomes (< 40%) have solid lines, those with high GC% (> 51%) have dotted lines, and those with intermediate GC% have broken lines.
