# Supplementary material for: Detection of horizontal transfer of individual genes by anomalous oligomer frequencies
Source: BMC Genomics. 2012 Jun 15;13:245. doi: 10.1186/1471-2164-13-245 (PMC3497702; doi:10.1186/1471-2164-13-245)
Supplement: Additional file 6 — Comparison of CGS method using hexamer or octamer frequencies. [file 1471-2164-13-245-S6.pdf]

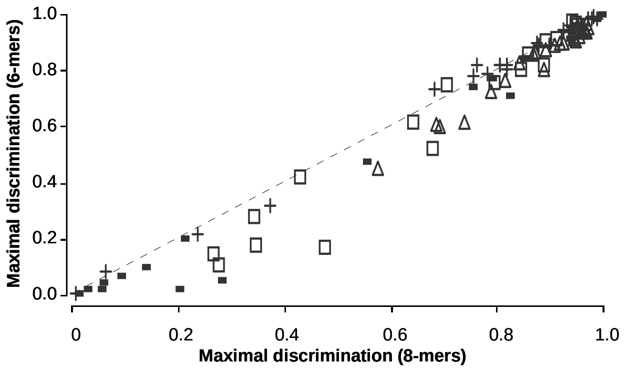

Additional File 4: Comparison of CGS method using hexamer or octamer frequencies. CGS scores were calculated using as targets the genomes of (□) *Ana*, (Δ) *Mar*, (+) *Pma*, and (–) *Pmt* and contaminating them to a level of 9% with genes from up to 25 different organisms (see Additional File 7).
