# Supplementary material for: Detection of horizontal transfer of individual genes by anomalous oligomer frequencies
Source: BMC Genomics. 2012 Jun 15;13:245. doi: 10.1186/1471-2164-13-245 (PMC3497702; doi:10.1186/1471-2164-13-245)
Supplement: Additional file 7 — Comparison of covariance and G-score methods. [file 1471-2164-13-245-S7.pdf]

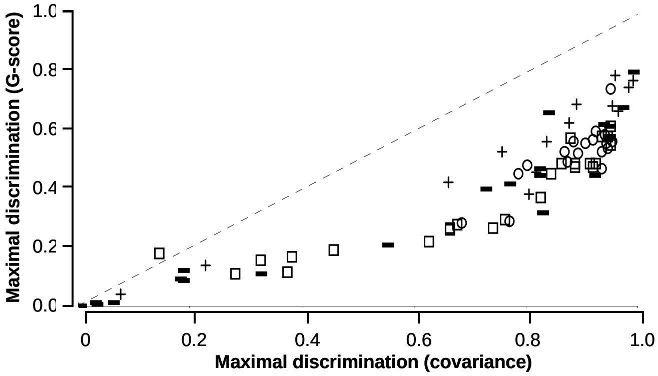

Additional File 5: Comparison of covariance and G-score methods. CGS scores were calculated using either covariance or G-score and using as targets the genomes of (□) *Ana*, (○) *Syn*, (+) *Pma*, and (–) *Pmt*, contaminating them to a level of 3% with genes from up to 25 different organisms (see Additional File 7).
