# Supplementary material for: Detection of horizontal transfer of individual genes by anomalous oligomer frequencies
Source: BMC Genomics. 2012 Jun 15;13:245. doi: 10.1186/1471-2164-13-245 (PMC3497702; doi:10.1186/1471-2164-13-245)
Supplement: Additional file 8 — Influence of size of reference set on maximal discrimination. [file 1471-2164-13-245-S8.pdf]

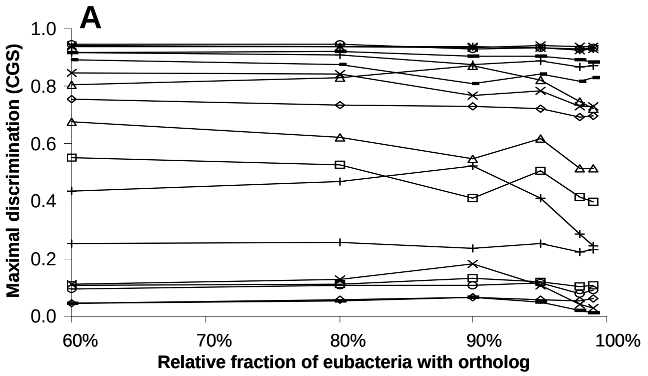

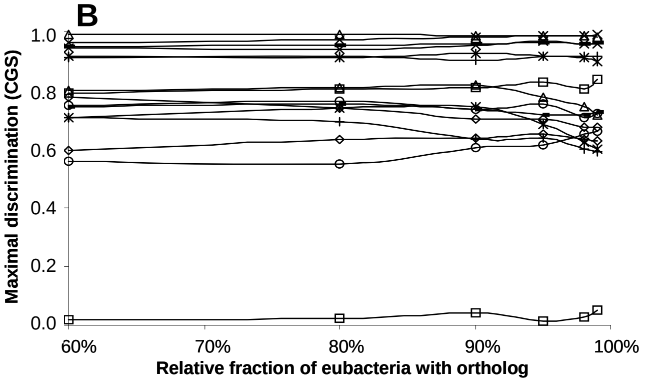

Additional File 6: Influence of size of reference set on maximal discrimination. CGS scores were calculated using reference sets composed of different numbers of conserved genes. The genes were selected to be in the reference set if the relative fraction of eubacteria in the KEGG data set bearing an ortholog of the gene exceeded a given threshold. The relative fraction is the number of eubacteria with an ortholog relative to the number of eubacteria with an ortholog to the most common gene. Using a dataset of 717 eubacteria, the highest number of orthologs found was 702, which considered 100%. Of the 753 genes in the set of core cyanobacterial genes, with *Syn* as the reference organism (see **Methods**):

	Threshold (relative fraction of eubacteria)							
	0%	60%	80%	90%	95%	98%	99%	100%
Number of genes of cyanobacterial core found at given threshold	753	464	313	217	154	106	80	2

(**A**) Maximal discrimination values were calculated by the CGS method, contaminating the set of *Ana* genes to a level of 9% with genes from various genomes. (**B**) Maximal discrimination values were calculated by the CGS method, contaminating the genes from various genomes to a level of 9% with genes from *Syp*. The outlying genome (□) is *Amar* (see Additional File 6).
