# Supplementary material for: Detection of horizontal transfer of individual genes by anomalous oligomer frequencies
Source: BMC Genomics. 2012 Jun 15;13:245. doi: 10.1186/1471-2164-13-245 (PMC3497702; doi:10.1186/1471-2164-13-245)
Supplement: Additional file 9 — Putative foreign gene summary in genomes without artificial contamination by foreign genes. [file 1471-2164-13-245-S9.pdf]

Additional File 7: Putative foreign gene summary in genomes without artificial contamination by foreign genes

Organism[a]	Genes[b]	Bulk genes[c]	Bulk Tns[d]	CGS %Foreign[e]	CGS %Foreign Tns[f]	GC %Foreign[e]	GC %Foreign Tns[f]	W8 %Foreign (orig)[g]	W8 %Foreign[e]	W8 %Foreign Tns[f]	Codon %Foreign (orig)[g]	Codon %Foreign[e]	Codon %Foreign Tns[f]
Ana	6132	5403	145	6.9 (1.9)	15.9	8.0 (3.0)	4.1	19.4	4.5 (-0.5)	8.3	7.6	14.0 (9.0)	6.9
Amar	8383	7655	239	24.9 (19.9)	20.5	9.8 (4.8)	0.4	17.4	10.9 (5.9)	2.9	7.0	28.4 (23.4)	5.9
Cya	2757	2033	90	24.2 (19.2)	97.8	11.3 (6.3)	36.7	17.4	21.3 (16.3)	92.2	6.3	23.1 (18.1)	47.8
Cyr	4390	3654	95	11.6 (6.6)	35.8	4.5 (-0.5)	4.2	22.1	5.1 (0.1)	3.2	7.9	19.9 (14.9)	20.0
Gvi	4430	3708	74	24.2 (19.2)	71.6	10.0 (5.0)	39.2	11.1	23.4 (18.4)	79.7	7.3	19.8 (14.8)	41.9
Lyng	6142	5657	197	8.5 (3.5)	25.9	6.9 (1.9)	0.0	23.0	5.8 (0.8)	11.2	not done	not done	
Mar	6312	5566	610	38.2 (33.2)	63.5	6.0 (1.0)	13.0	15.0	11.8 (6.8)	2.5	3.7	38.4 (33.4)	14.1
Npu	7321	6602	359	5.4 (0.4)	6.4	8.6 (3.6)	10.3	19.9	3.2 (-1.8)	0.8	12.0	12.8 (7.8)	15.9
Pmt	2267	1488	0	22.4 (17.4)		9.6 (4.6)		13.0	16.5 (11.5)		6.6	22.1 (17.1)	
Pmm	1713	930	0	6.8 (1.8)		7.6 (2.6)		20.4	5.9 (0.9)		7.1	20.8 (15.8)	
Pmf	2997	2220	0	27.7 (22.7)		9.1 (4.1)		11.7	17.3 (12.3)		4.1	40.9 (35.9)	
Pmn	1892	1122	0	7.8 (2.8)		8.9 (3.9)		20.2	8.1 (3.1)		8.1	22.8 (17.8)	
Pmz	2123	1347	5	21.6 (16.6)	100.0	18.0 (13.0)	100.0	14.2	20.4 (15.4)	100.0	not done	not done	
Pma	1882	1084	0	6.9 (1.9)		7.0 (2.0)		18.0	7.6 (2.6)		9.3	26.4 (21.4)	
Syn	3672	2919	127	26.3 (21.3)	98.4	16.2 (11.2)	58.3	6.3	16.8 (11.8)	38.6	9.8	16.4 (11.4)	58.3
Syf	2669	1900	1	17.2 (12.2)	0.0	14.1 (9.1)	0.0	11.3	11.7 (6.7)	100.0	8.3	16.7 (11.7)	0.0
Syw	2526	1749	0	27.3 (22.2)		15.6 (10.6)		11.8	22.1 (17.1)		3.9	32.1 (27.1)	
Syd	2637	1867	0	32.5 (27.5)		17.6 (12.6)		10.1	29.0 (24.0)		4.3	33.9 (28.9)	
Sye	2304	1522	0	22.7 (17.7)		14.1 (9.1)		15.6	21.9 (16.9)		7.1	25.0 (20.0)	
Syg	2892	2119	0	31.8 (26.8)		12.8 (7.8)		11.3	20.4 (15.3)		5.4	33.6 (28.6)	
Syp	3186	2433	10	20.5 (15.5)	90.0	14.3 (9.3)	0.0	4.1	10.9 (5.9)	90.0	8.0	15.4 (10.4)	0.0
Syx	2533	1761	0	17.5 (12.5)		11.6 (6.6)		14.5	14.4 (9.4)		7.6	22.6 (17.6)	
Tel	2475	1710	82	18.9 (13.9)	95.1	12.0 (7.0)	50.0	10.0	16.4 (11.4)	69.5	9.9	11.8 (6.8)	29.3
Ter	4451	3706	165	8.3 (3.3)	7.9	10.1 (5.1)	1.2	14.4	6.1 (1.1)	9.7	9.5	15.3 (10.3)	39.4
Mean				19.2 (14.2)		11.0 (6.0)		14.7	13.8 (8.8)		7.3	23.3 (18.3)	
Std Dev				9.5		3.7		4.9	7.2		2.1	8.3	

[a] Full names of organisms are given in Fig. 1
[b] Number of predicted protein-encoding genes in the genome, including sequences of plasmids
[c] Number of predicted protein-encoding genes, less genes in core and conserved genes used as the test-native set
[d] Number of genes annotated as transposases
[e] Fraction of protein-encoding genes predicted by method to be foreign, using a 5% threshold (see text for explanation). The number in parentheses is the fraction predicted in excess of the expected 5%.
[f] Fraction of genes annotated as transposases found amongst those predicted to be of foreign origin.
[g] Fraction of protein-encoding genes predicted by method to be foreign, using the method suggested by Tsirigos and Rigoutsos (2005) for W8 and by Mrazek et al (2001) for codon bias.
