# Supplementary material for: Detection of horizontal transfer of individual genes by anomalous oligomer frequencies
Source: BMC Genomics. 2012 Jun 15;13:245. doi: 10.1186/1471-2164-13-245 (PMC3497702; doi:10.1186/1471-2164-13-245)

Analysis of phylogenetic conflicts from Zhaxybayeva et al (2006)

Phylogenetic conflicts observed by Zhaxybayeva et al (2006)* were analyzed as shown in the pages that follow. 12 of these (**identifiers shown in green**) were selected because the discordant genes included one with a CGS score < 0.05. The other 9 conflicts (**identifiers shown in red**) were selected at random. In addition, trees are provided for proteins similar to 4 randomly chosen proteins from *Synechocystis* with CGS scores < 0.05 (**identifiers shown in blue**) .

Trees were constructed by a Bayesian approach (left tree) or maximum likelihood (right tree) as described in **Methods**.

<u>CGS scores</u>

The percentage of test core genes with worse raw CGS scores than the gene in question. Scores 5 or below are highlighted.

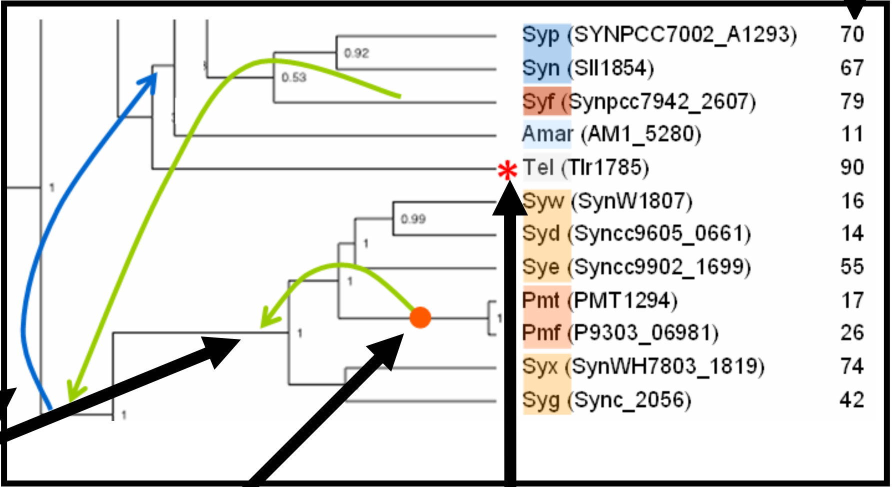

Transposition of a gene as shown by an arrow would make the gene concordant with the 16S RNA tree. Green arrows indicate a transposition not well supported by the maximum likelihood tree.

Indicates discordance between the Bayesian and the maximum likelihood tree.

Genes whose relationship was deemed by quartet analysis (Zhaxybayeva et al, 2006) to be discordant with the 16S-RNA tree

*Zhaxybayeva O et al (2006). Genome Res 16:1099-1108

16S RNA Tree used as a basis to judge discordances

The 16S RNA tree shown to the right was taken from Fig. 1, constructed as described in **Methods**.

The color conventions to denote organismal groups in the are maintained in the pages that follow.

Names (e.g. PMT1204) following the organism abbreviations indicate the genes used to construct the tree.

Asterisks mark the organisms whose genomes were considered by Zhaxybayeva et al (2006). Conflicts in trees marked with asterisks were based solely on these organisms.

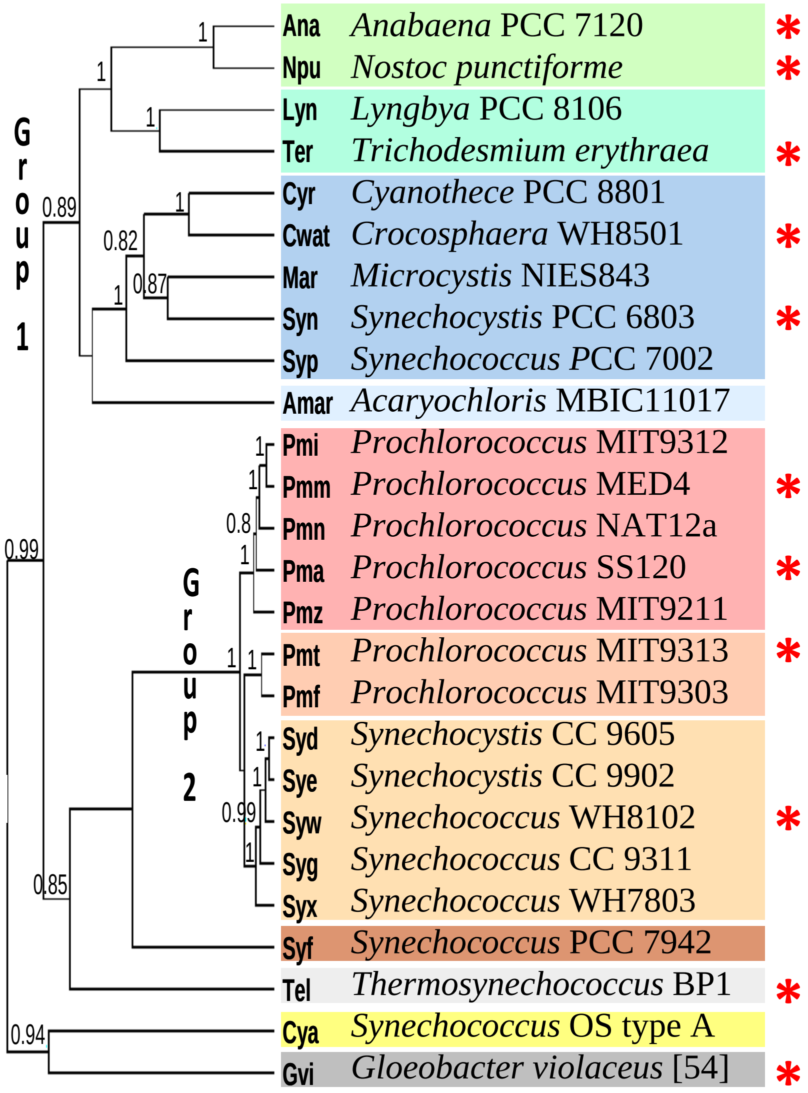

Zh162

RbcS (109 aa)

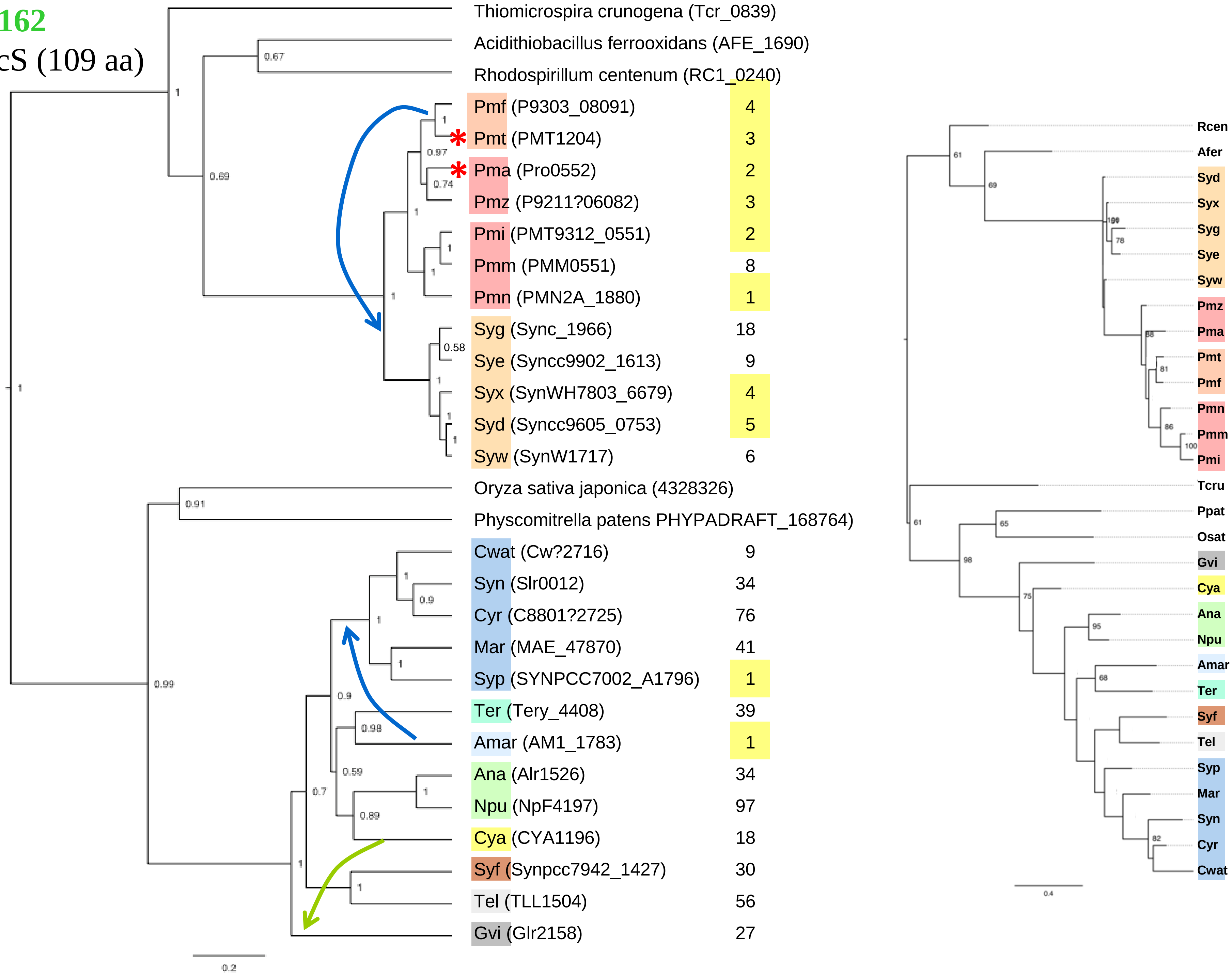

Zh173
hypothetical
(84 aa)

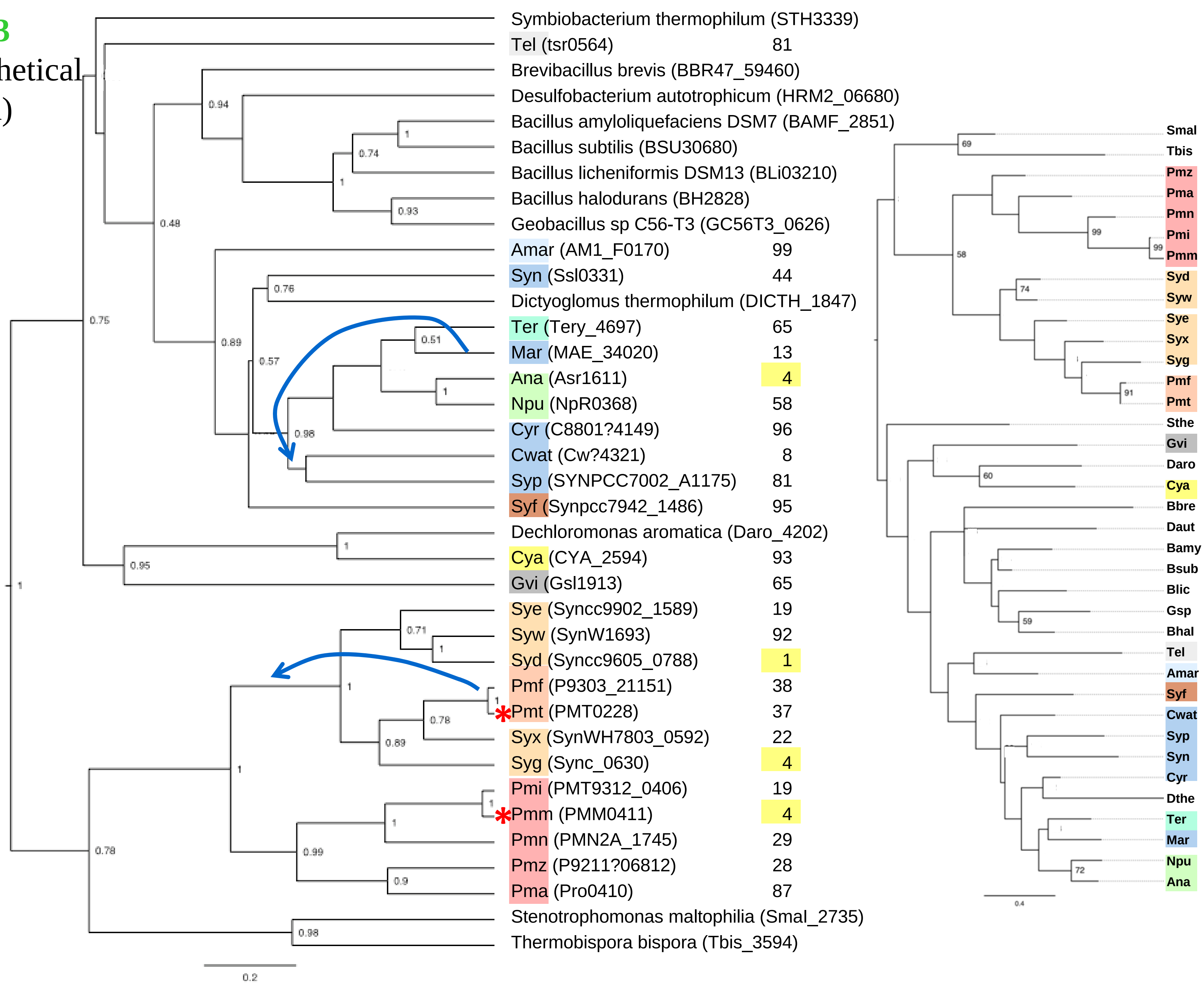

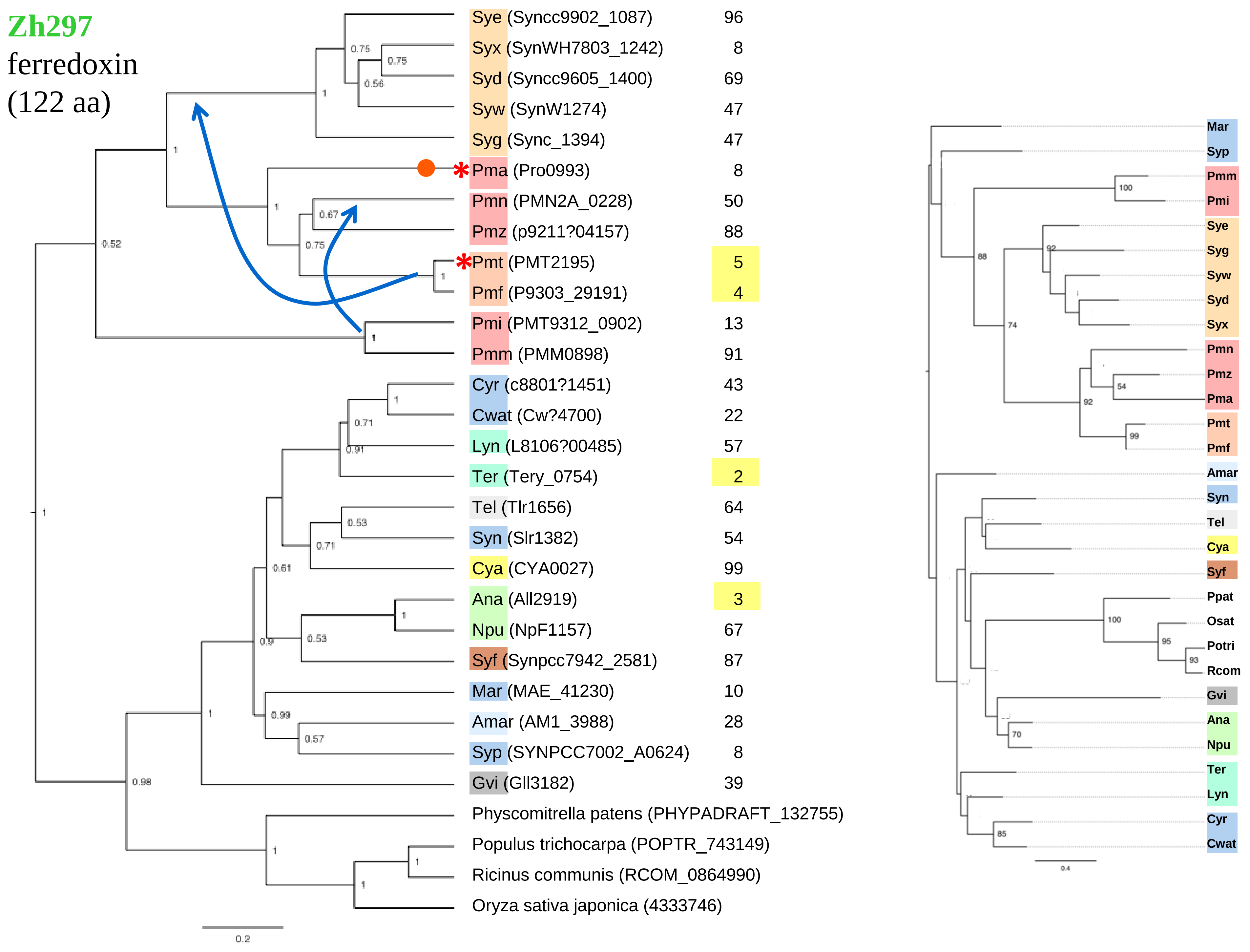

Zh297
ferredoxin
(122 aa)
Sye (Syncc9902_1087) 96
Syx (SynWH7803_1242) 8
Syd (Syncc9605_1400) 69
Syw (SynW1274) 47
Syg (Sync_1394) 47
Pma (Pro0993) 8
Pmn (PMN2A_0228) 50
Pmz (p9211?04157) 88
Pmt (PMT2195) 5
Pmf (P9303_29191) 4
Pmi (PMT9312_0902) 13
Pmm (PMM0898) 91
Cyr (c8801?1451) 43
Cwat (Cw?4700) 22
Lyn (L8106?00485) 57
Ter (Tery_0754) 2
Tel (Tlr1656) 64
Syn (Slr1382) 54
Cya (CYA0027) 99
Ana (All2919) 3
Npu (NpF1157) 67
Syf (Synpcc7942_2581) 87
Mar (MAE_41230) 10
Amar (AM1_3988) 28
Syp (SYNPCC7002_A0624) 8
Gvi (Gll3182) 39
Physcomitrella patens (PHYPADRAFT_132755)
Populus trichocarpa (POPTR_743149)
Ricinus communis (RCOM_0864990)
Oryza sativa japonica (4333746)
0.2
Mar
Syp
Pmm
Pmi
Sye
Syg
Syw
Syd
Syx
Pmn
Pmz
Pma
Pmt
Pmf
Amar
Syn
Tel
Cya
Syf
Ppat
Osat
Potri
Rcom
Gvi
Ana
Npu
Ter
Lyn
Cyr
Cwat
0.4

Zh300

hypothetical

(107 aa)

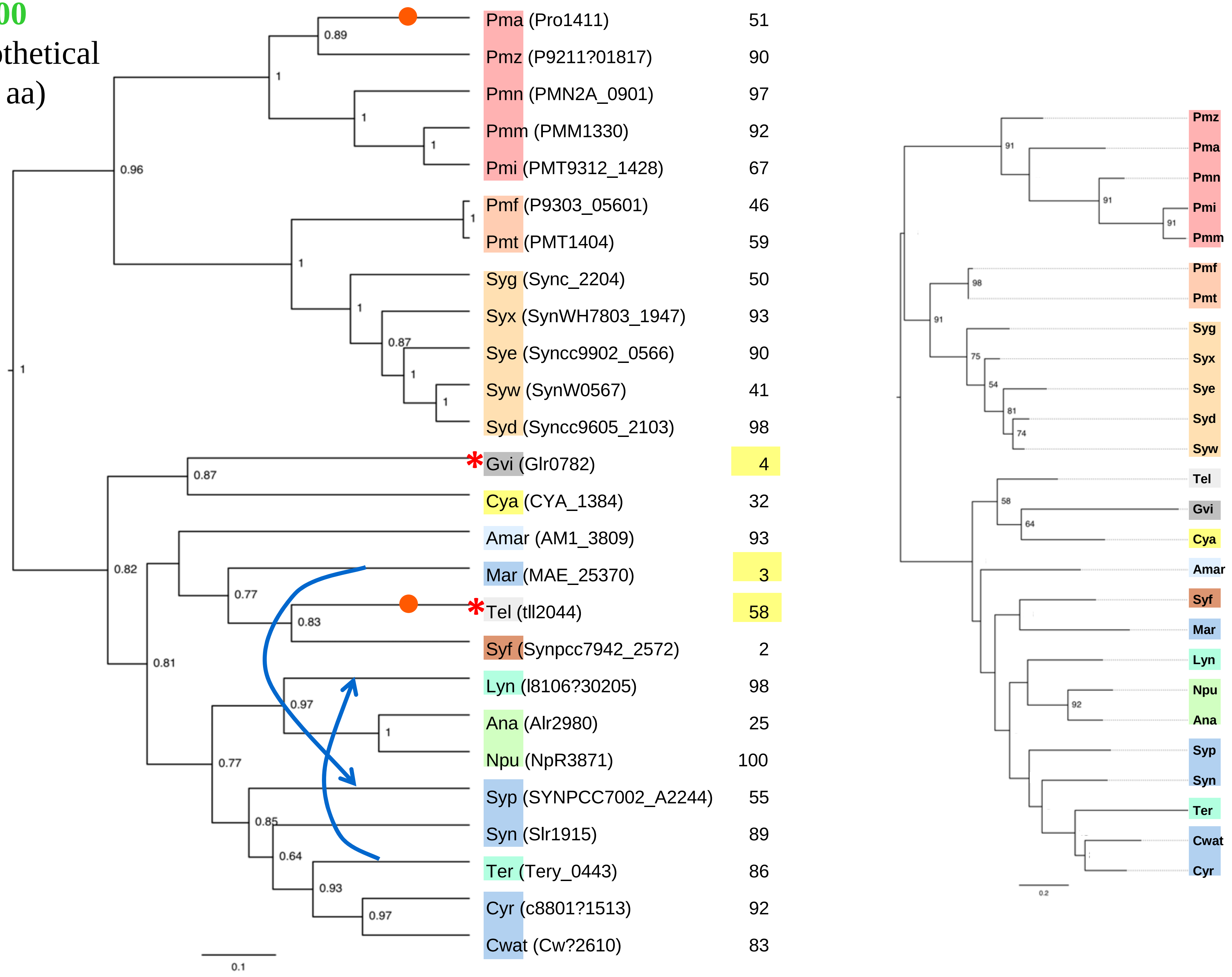

Zh312

sugar fermentation stimulation (241 aa)

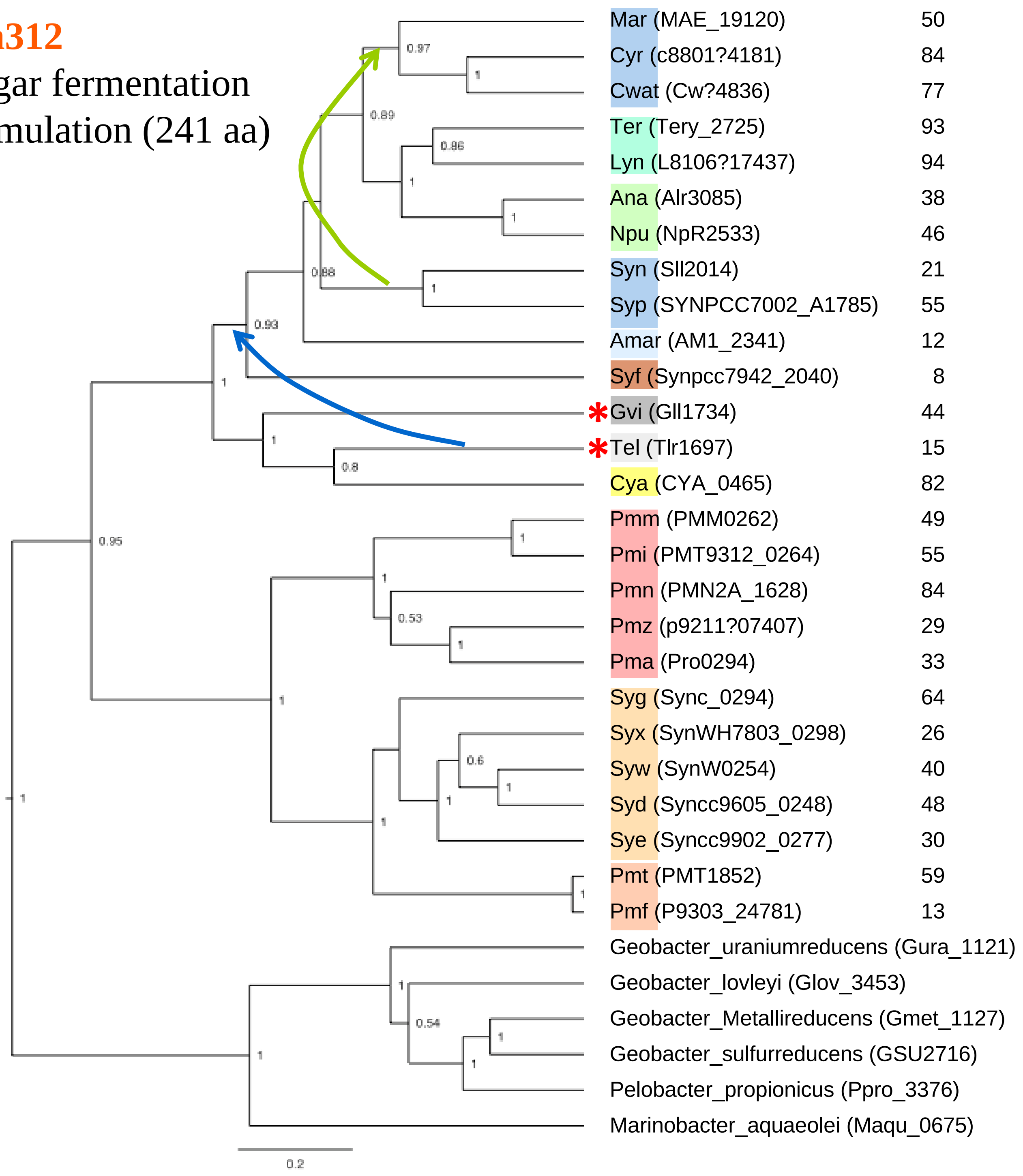

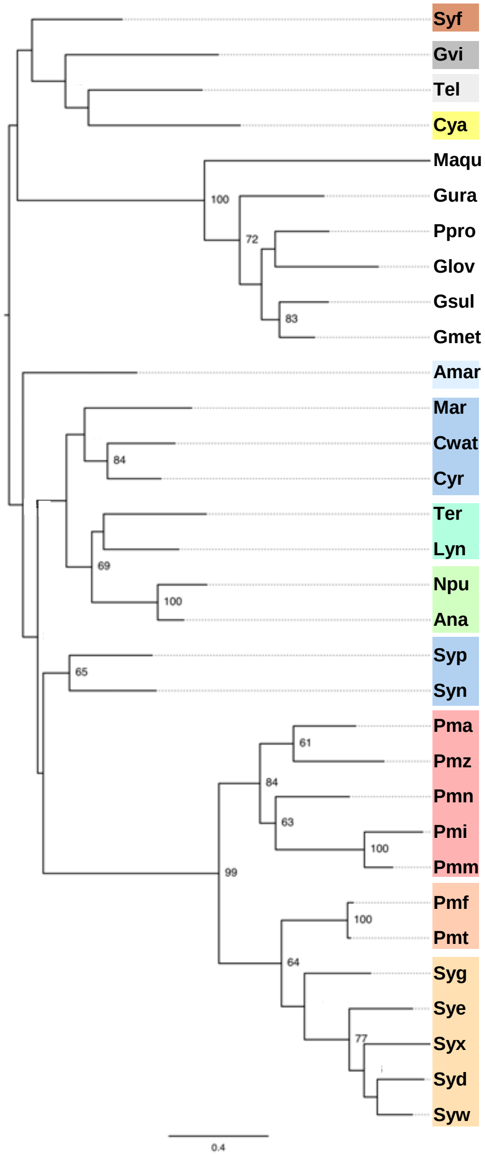

Zh388

amidophospho-ribosyltransferase (499 aa)

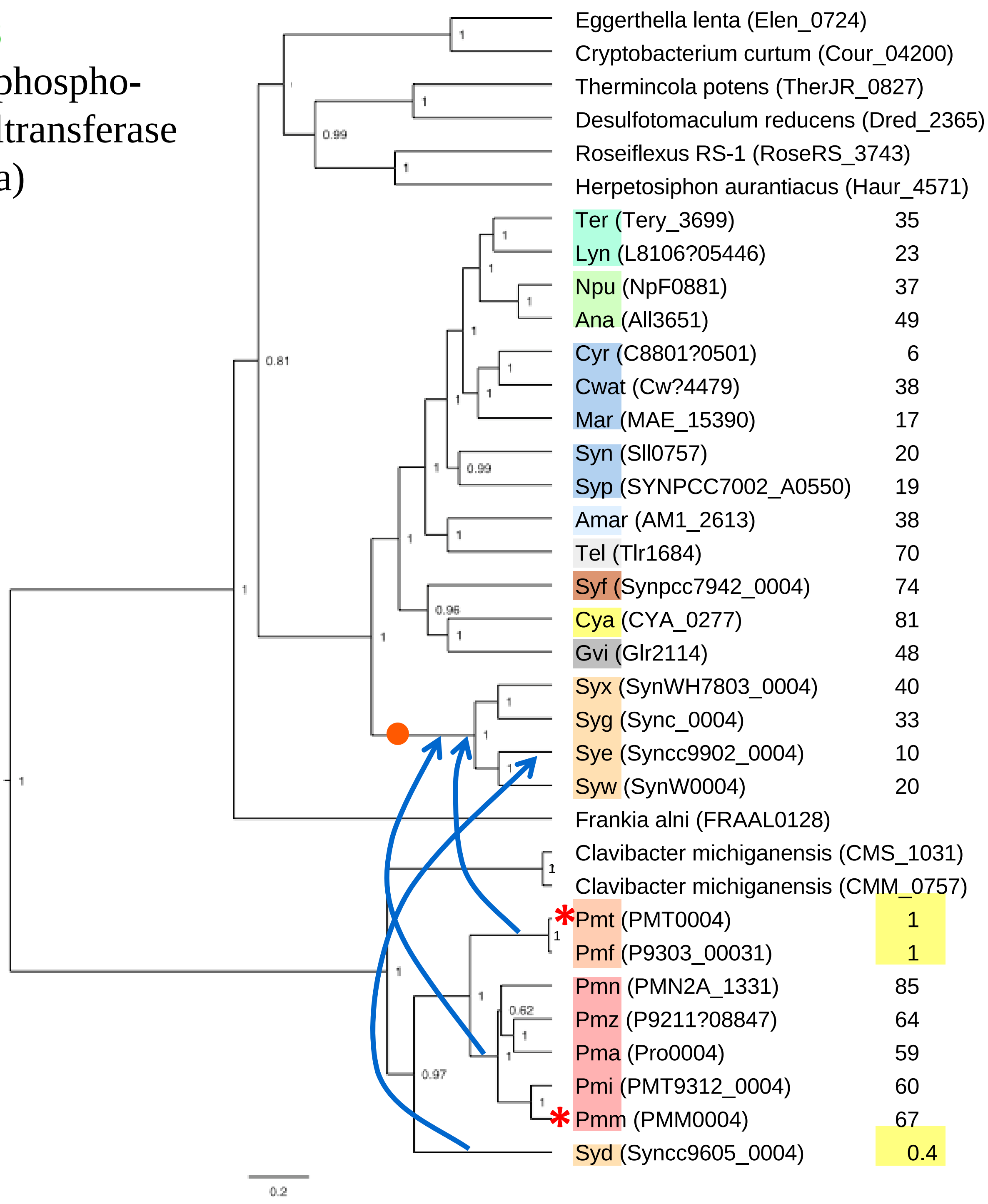

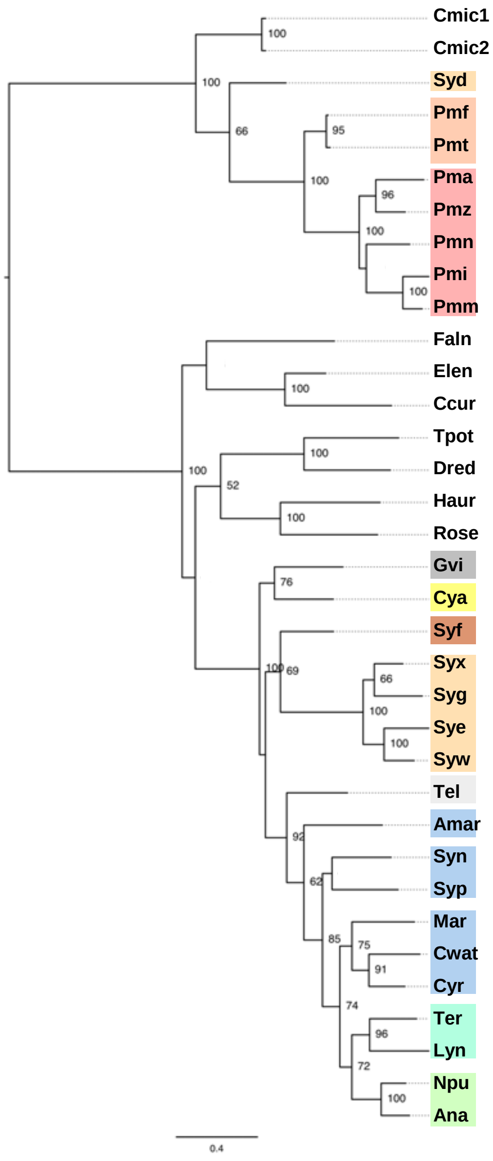

Zh632

transcription regulator?

(229 aa)

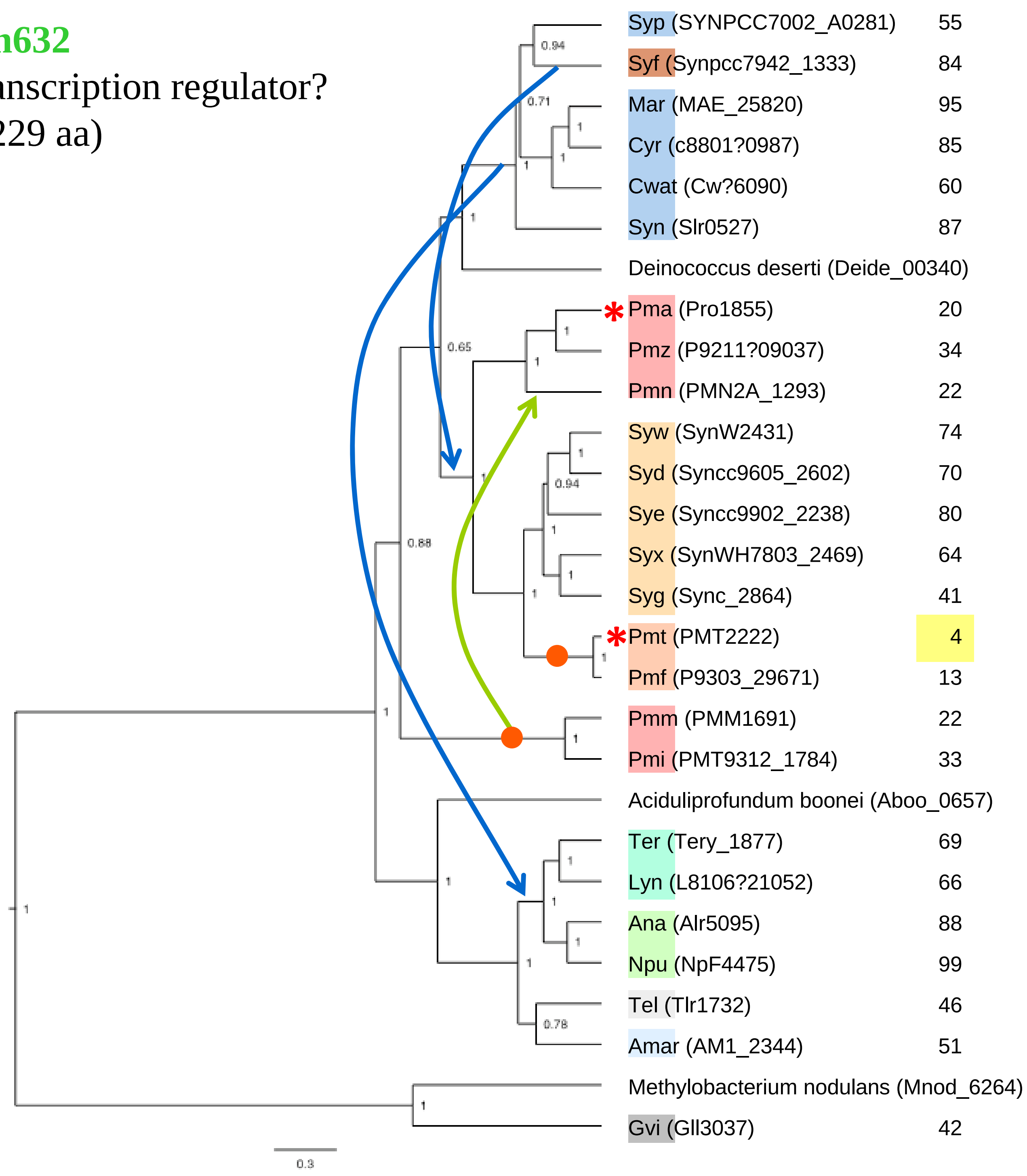

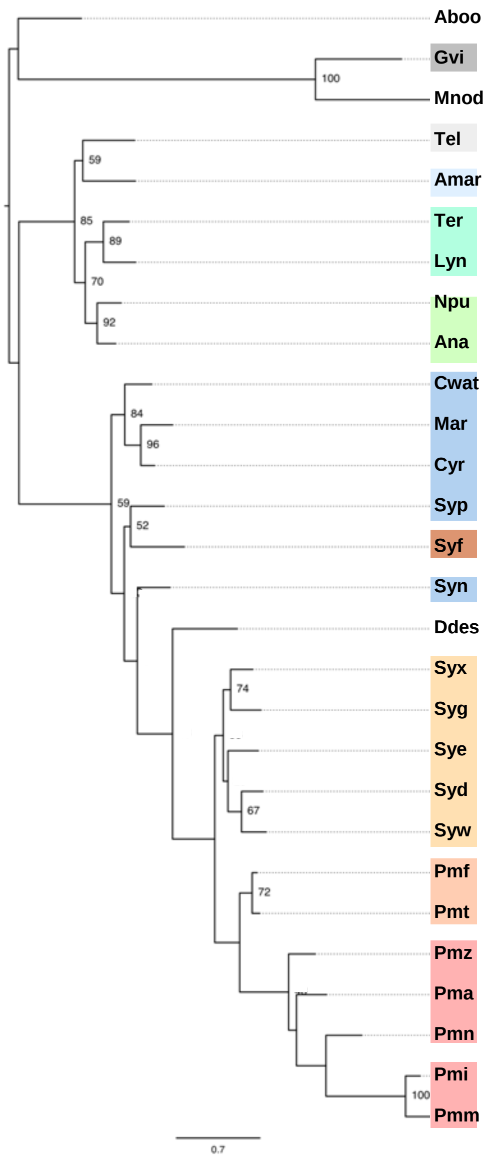

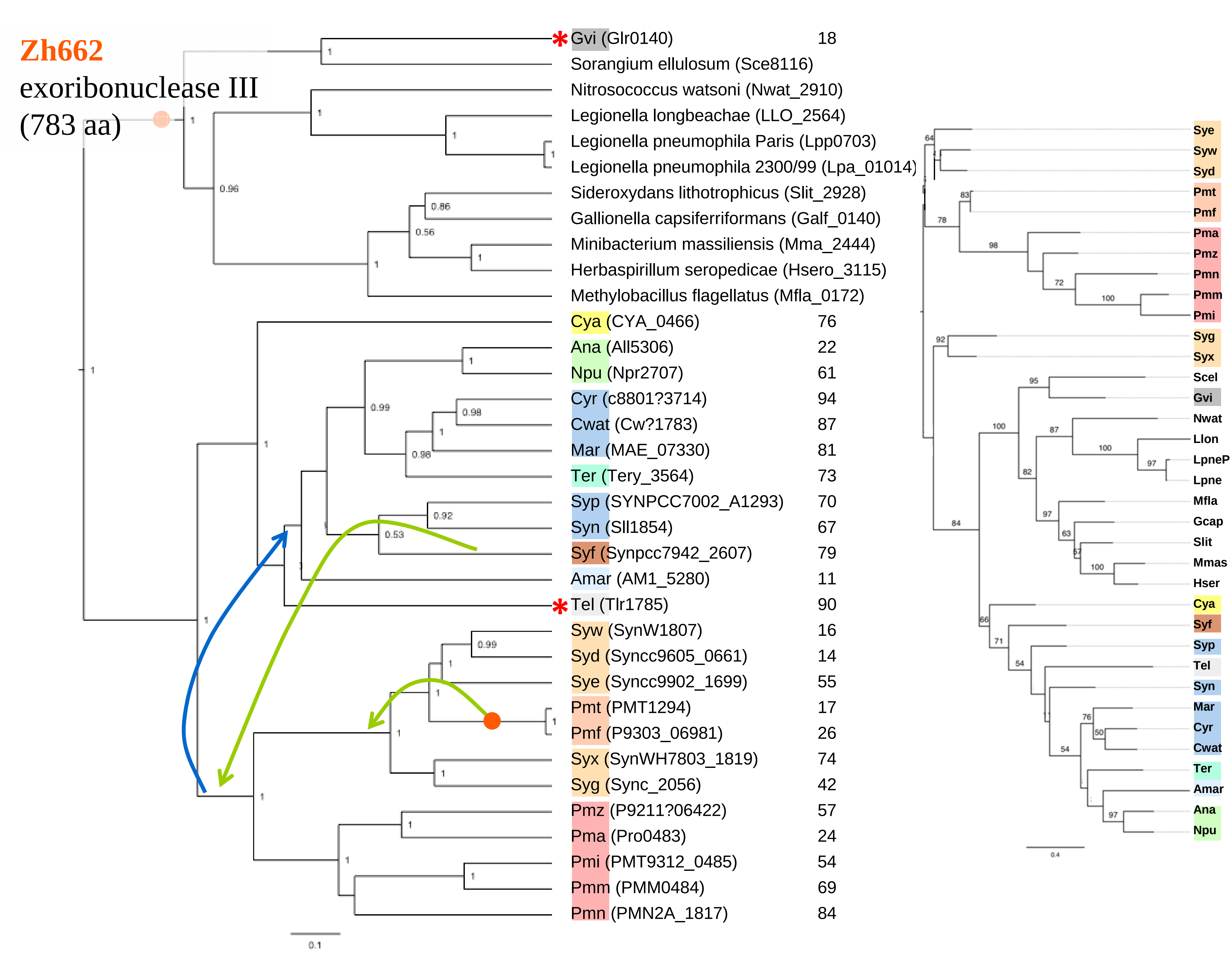

Zh662
exoribonuclease III
(783 aa)
Gvi (Glr0140) 18
Sorangium ellulosum (Sce8116)
Nitrosococcus watsoni (Nwat_2910)
Legionella longbeachae (LLO_2564)
Legionella pneumophila Paris (Lpp0703)
Legionella pneumophila 2300/99 (Lpa_01014)
Sideroxydans lithotrophicus (Slit_2928)
Gallionella capsiferriformans (Galf_0140)
Minibacterium massiliensis (Mma_2444)
Herbaspirillum seropedicae (Hsero_3115)
Methylobacillus flagellatus (Mfla_0172)
Cya (CYA_0466) 76
Ana (All5306) 22
Npu (Npr2707) 61
Cyr (c8801?3714) 94
Cwat (Cw?1783) 87
Mar (MAE_07330) 81
Ter (Tery_3564) 73
Syp (SYNPCC7002_A1293) 70
Syn (Sll1854) 67
Syf (Synpcc7942_2607) 79
Amar (AM1_5280) 11
Tel (Tlr1785) 90
Syw (SynW1807) 16
Syd (Syncc9605_0661) 14
Sye (Syncc9902_1699) 55
Pmt (PMT1294) 17
Pmf (P9303_06981) 26
Syx (SynWH7803_1819) 74
Syg (Sync_2056) 42
Pmz (P9211?06422) 57
Pma (Pro0483) 24
Pmi (PMT9312_0485) 54
Pmm (PMM0484) 69
Pmn (PMN2A_1817) 84
Sye
Syw
Syd
Pmt
Pmf
Pma
Pmz
Pmn
Pmm
Pmi
Syg
Syx
Scel
Gvi
Nwat
Llon
LpneP
Lpne
Mfla
Gcap
Slit
Mmas
Hser
Cya
Syf
Syp
Tel
Syn
Mar
Cyr
Cwat
Ter
Amar
Ana
Npu

Zh672

delta amino levulinic acid dehytratase (326 aa)

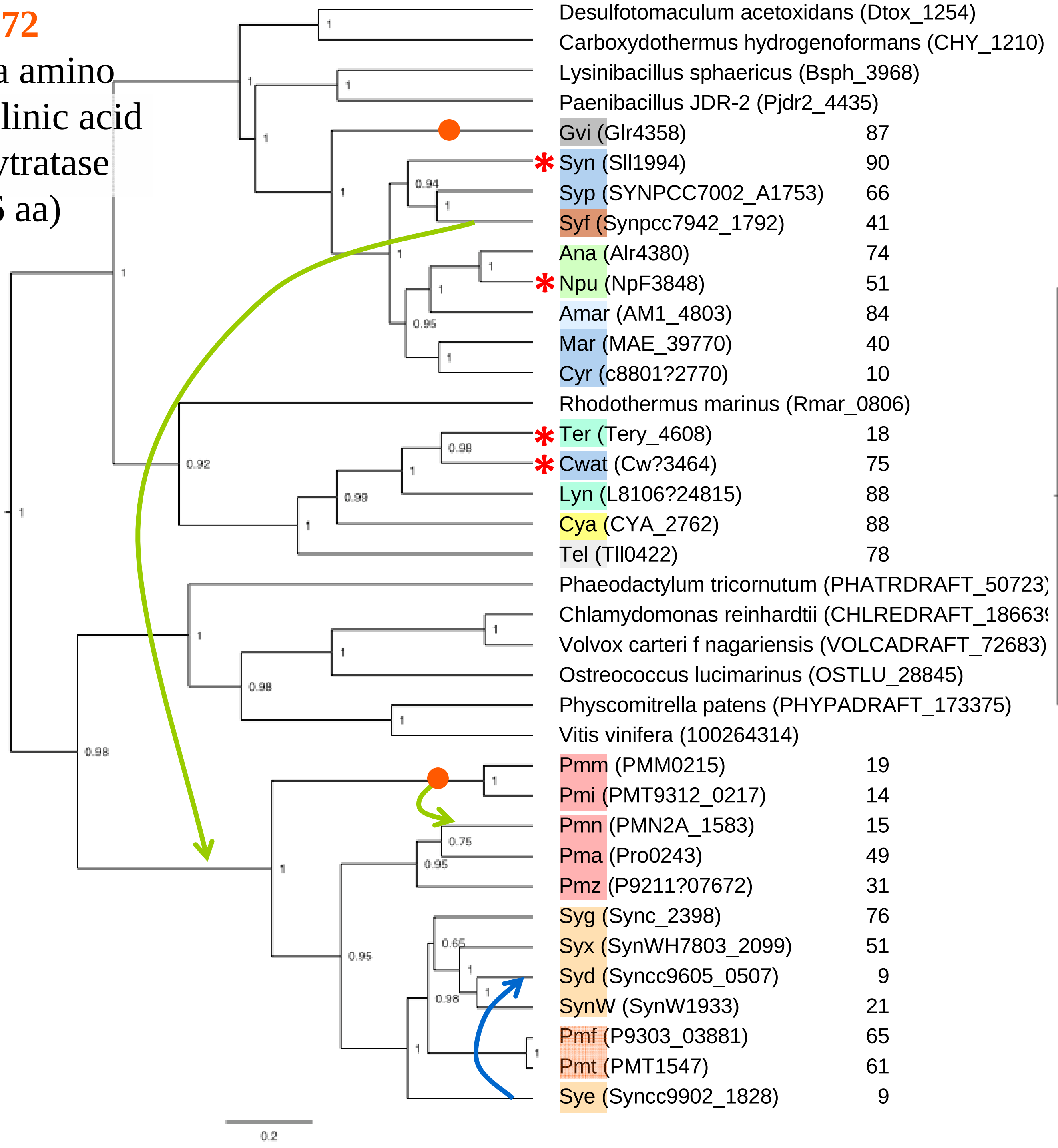

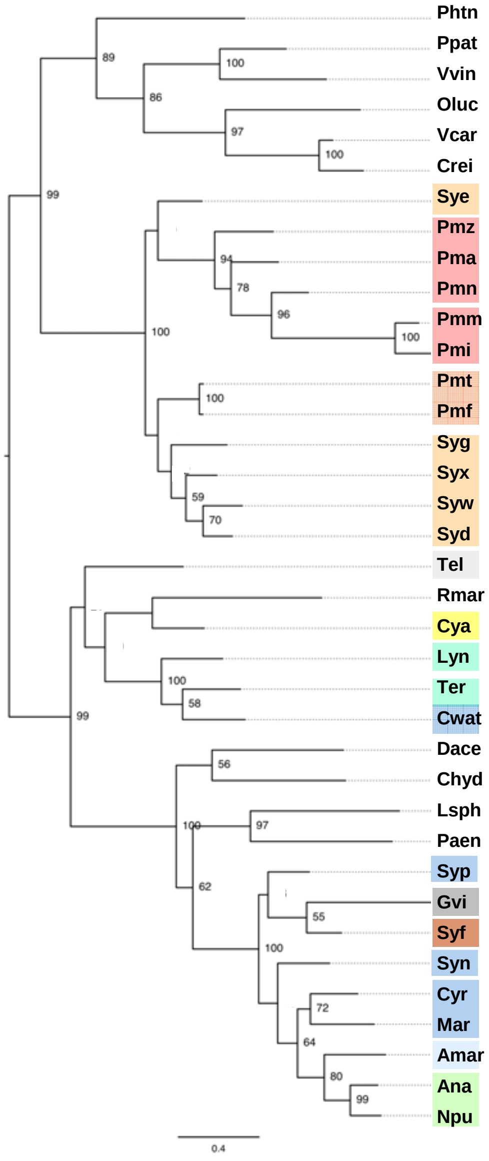

Zh685

hypothetical

(65 aa)

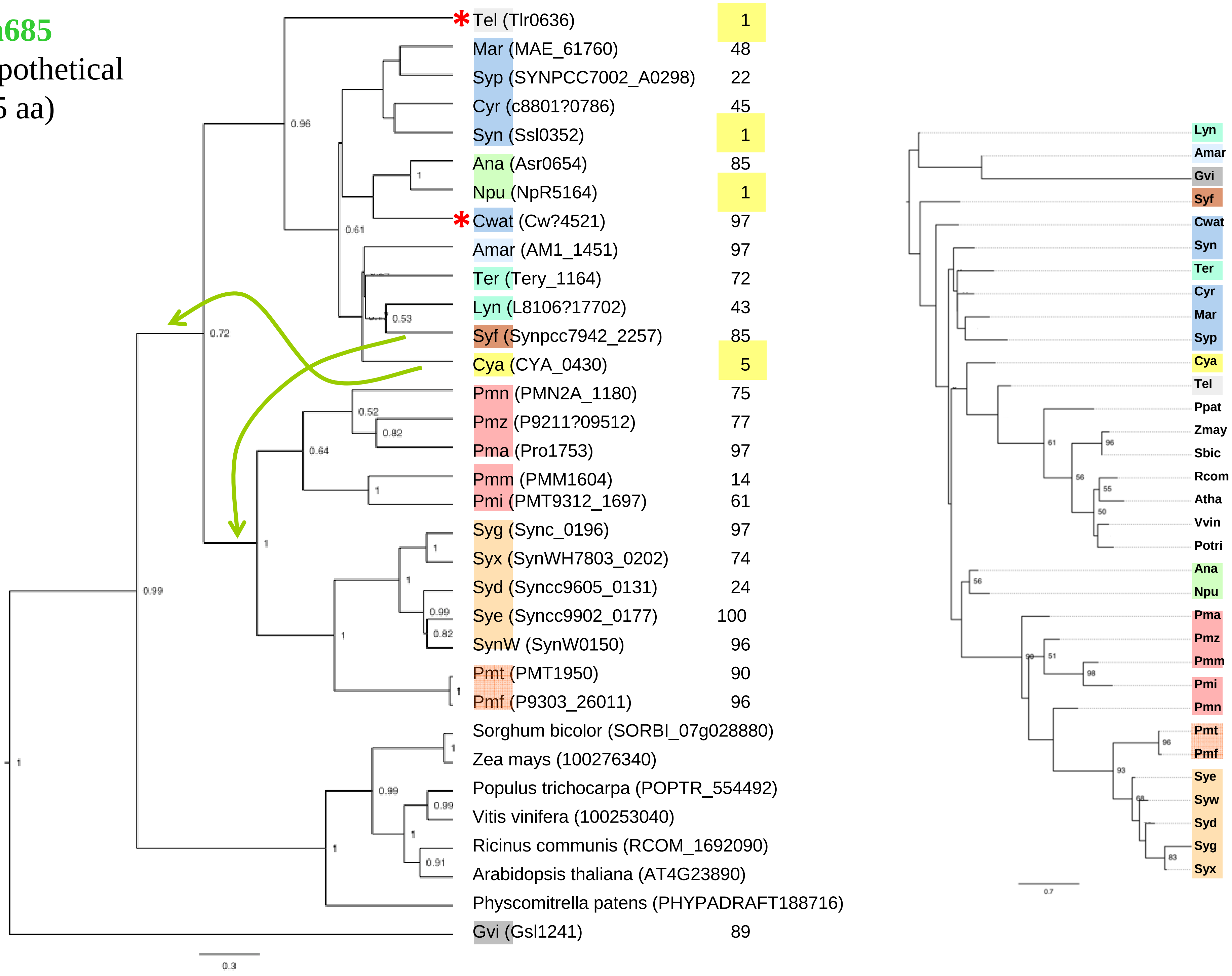

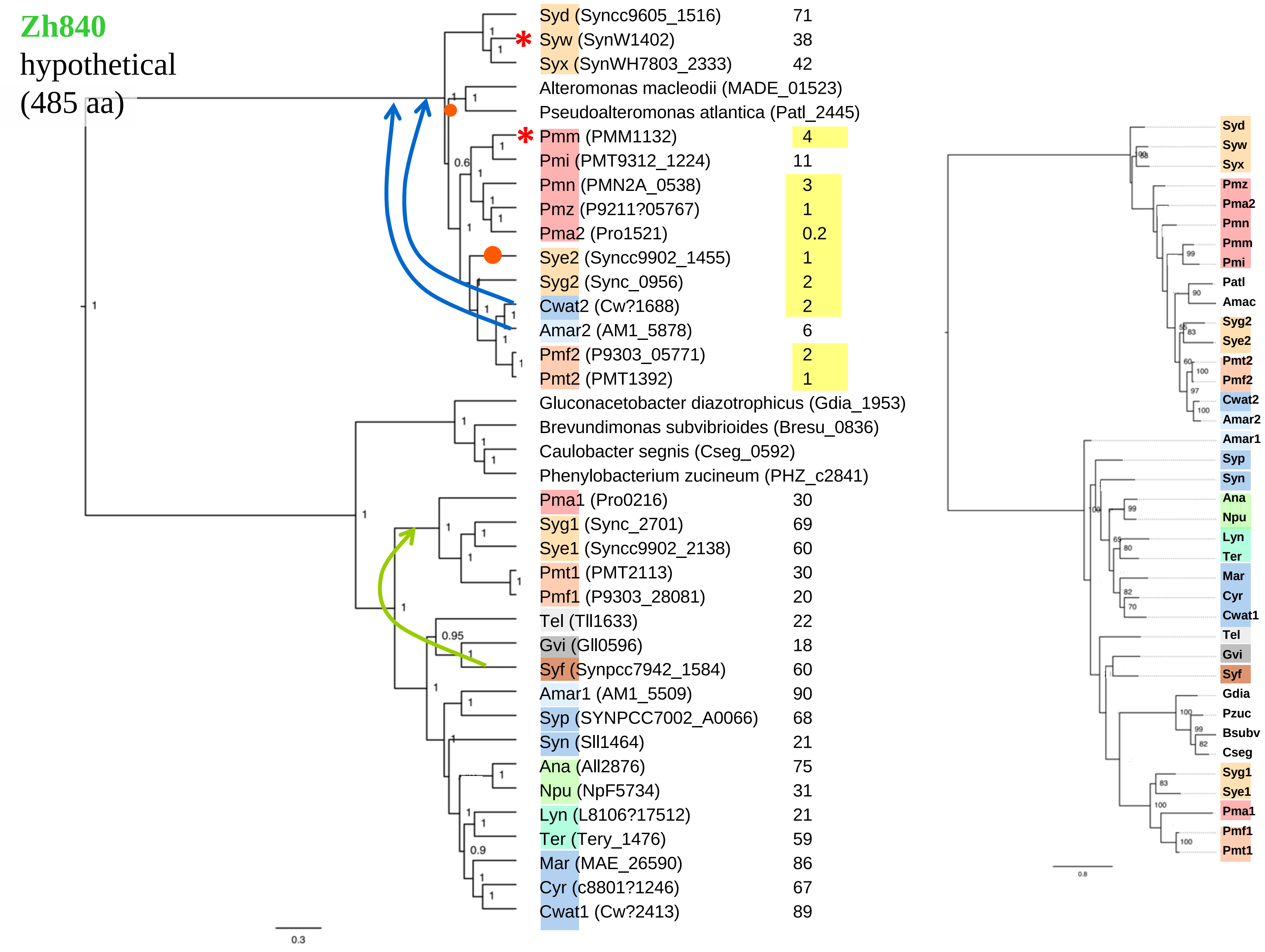

Zh840
hypothetical
(485 aa)
Syd (Syncc9605_1516) 71
* Syw (SynW1402) 38
Syx (SynWH7803_2333) 42
Alteromonas macleodii (MADE_01523)
Pseudoalteromonas atlantica (Patl_2445)
* Pmm (PMM1132) 4
Pmi (PMT9312_1224) 11
Pmn (PMN2A_0538) 3
Pmz (P9211?05767) 1
Pma2 (Pro1521) 0.2
Sye2 (Syncc9902_1455) 1
Syg2 (Sync_0956) 2
Cwat2 (Cw?1688) 2
Amar2 (AM1_5878) 6
Pmf2 (P9303_05771) 2
Pmt2 (PMT1392) 1
Gluconacetobacter diazotrophicus (Gdia_1953)
Brevundimonas subvibrioides (Bresu_0836)
Caulobacter segnis (Cseg_0592)
Phenylobacterium zucineum (PHZ_c2841)
Pma1 (Pro0216) 30
Syg1 (Sync_2701) 69
Sye1 (Syncc9902_2138) 60
Pmt1 (PMT2113) 30
Pmf1 (P9303_28081) 20
Tel (Tll1633) 22
Gvi (Gll0596) 18
Syf (Synpcc7942_1584) 60
Amar1 (AM1_5509) 90
Syp (SYNPCC7002_A0066) 68
Syn (Sll1464) 21
Ana (All2876) 75
Npu (NpF5734) 31
Lyn (L8106?17512) 21
Ter (Tery_1476) 59
Mar (MAE_26590) 86
Cyr (c8801?1246) 67
Cwat1 (Cw?2413) 89
0.6
0.95
0.9
0.3
Syd
Syw
Syx
Pmz
Pma2
Pmn
Pmm
Pmi
Patl
Amac
Syg2
Sye2
Pmt2
Pmf2
Cwat2
Amar2
Amar1
Syp
Syn
Ana
Npu
Lyn
Ter
Mar
Cyr
Cwat1
Tel
Gvi
Syf
Gdia
Pzuc
Bsubv
Cseg
Syg1
Sye1
Pma1
Pmf1
Pmt1
0.8

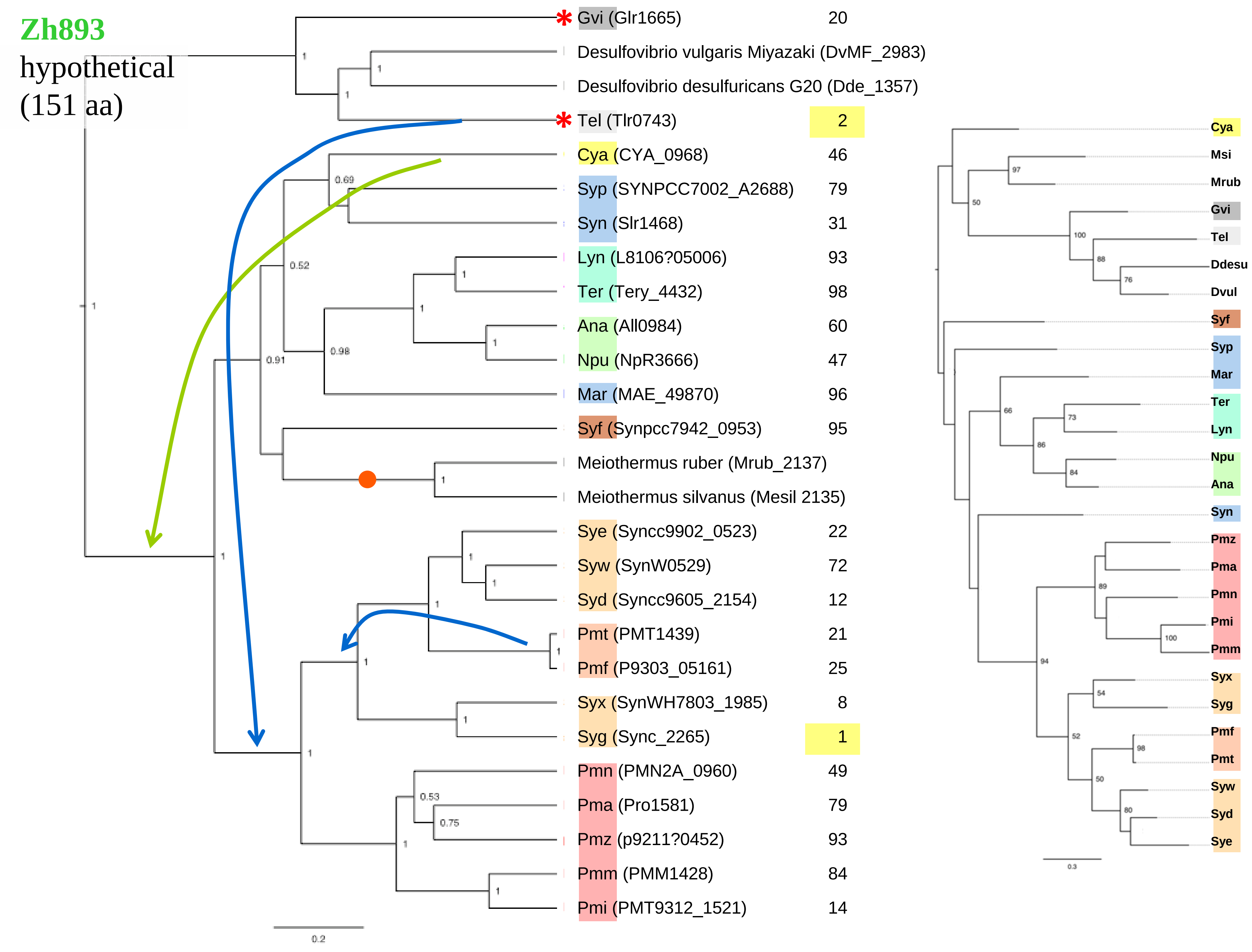
Zh893
hypothetical
(151 aa)
Gvi (Glr1665) 20
Desulfovibrio vulgaris Miyazaki (DvMF_2983)
Desulfovibrio desulfuricans G20 (Dde_1357)
Tel (Tlr0743) 2
Cya (CYA_0968) 46
Syp (SYNPCC7002_A2688) 79
Syn (Slr1468) 31
Lyn (L8106?05006) 93
Ter (Tery_4432) 98
Ana (All0984) 60
Npu (NpR3666) 47
Mar (MAE_49870) 96
Syf (Synpcc7942_0953) 95
Meiothermus ruber (Mrub_2137)
Meiothermus silvanus (Mesil 2135)
Sye (Syncc9902_0523) 22
Syw (SynW0529) 72
Syd (Syncc9605_2154) 12
Pmt (PMT1439) 21
Pmf (P9303_05161) 25
Syx (SynWH7803_1985) 8
Syg (Sync_2265) 1
Pmn (PMN2A_0960) 49
Pma (Pro1581) 79
Pmz (p9211?0452) 93
Pmm (PMM1428) 84
Pmi (PMT9312_1521) 14
0.2
Cya
Msi
Mrub
Gvi
Tel
Ddesu
Dvul
Syf
Syp
Mar
Ter
Lyn
Npu
Ana
Syn
Pmz
Pma
Pmn
Pmi
Pmm
Syx
Syg
Pmf
Pmt
Syw
Syd
Sye
0.3

Zh907

Sterol methyltransferase (330 aa)

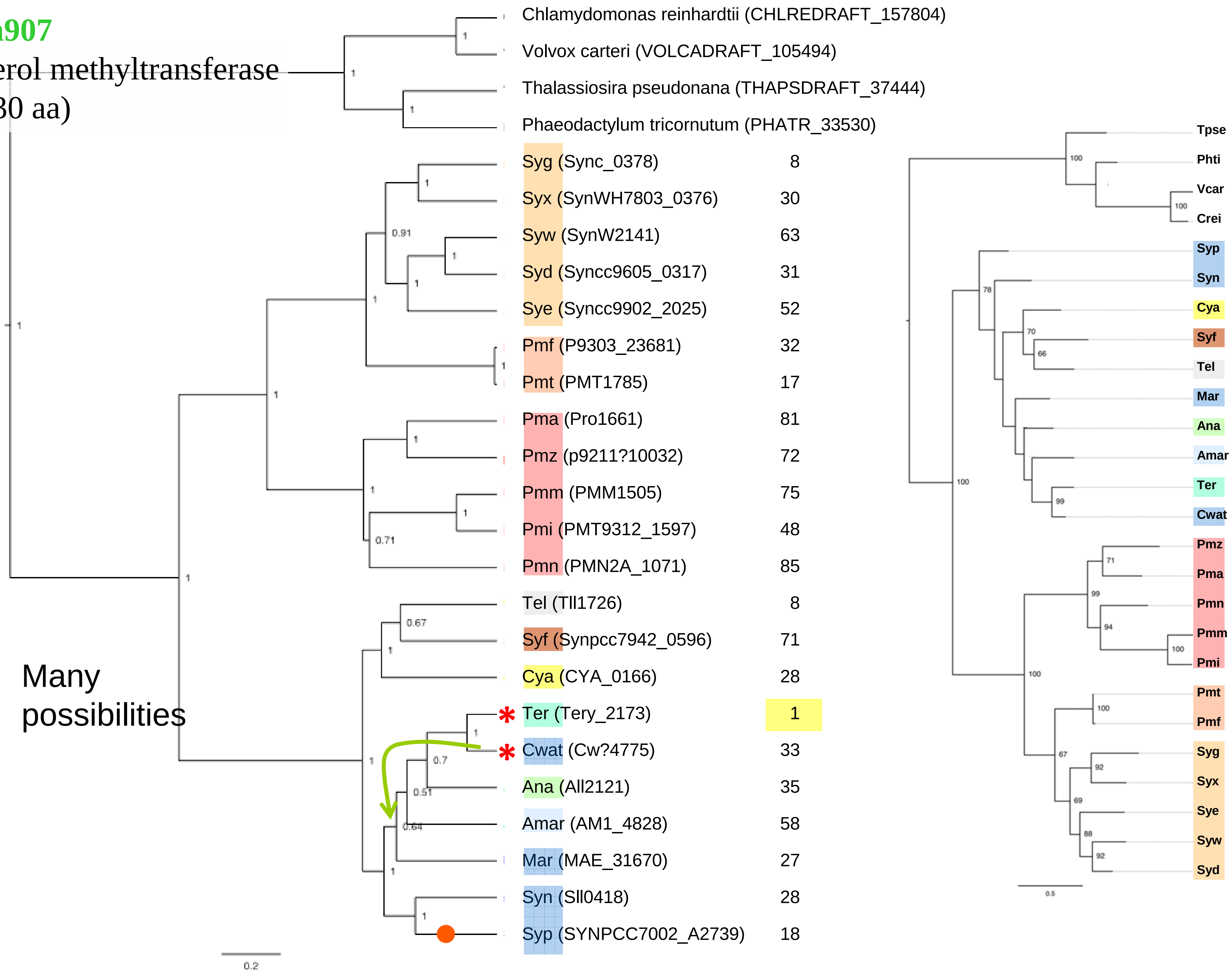

Many possibilities

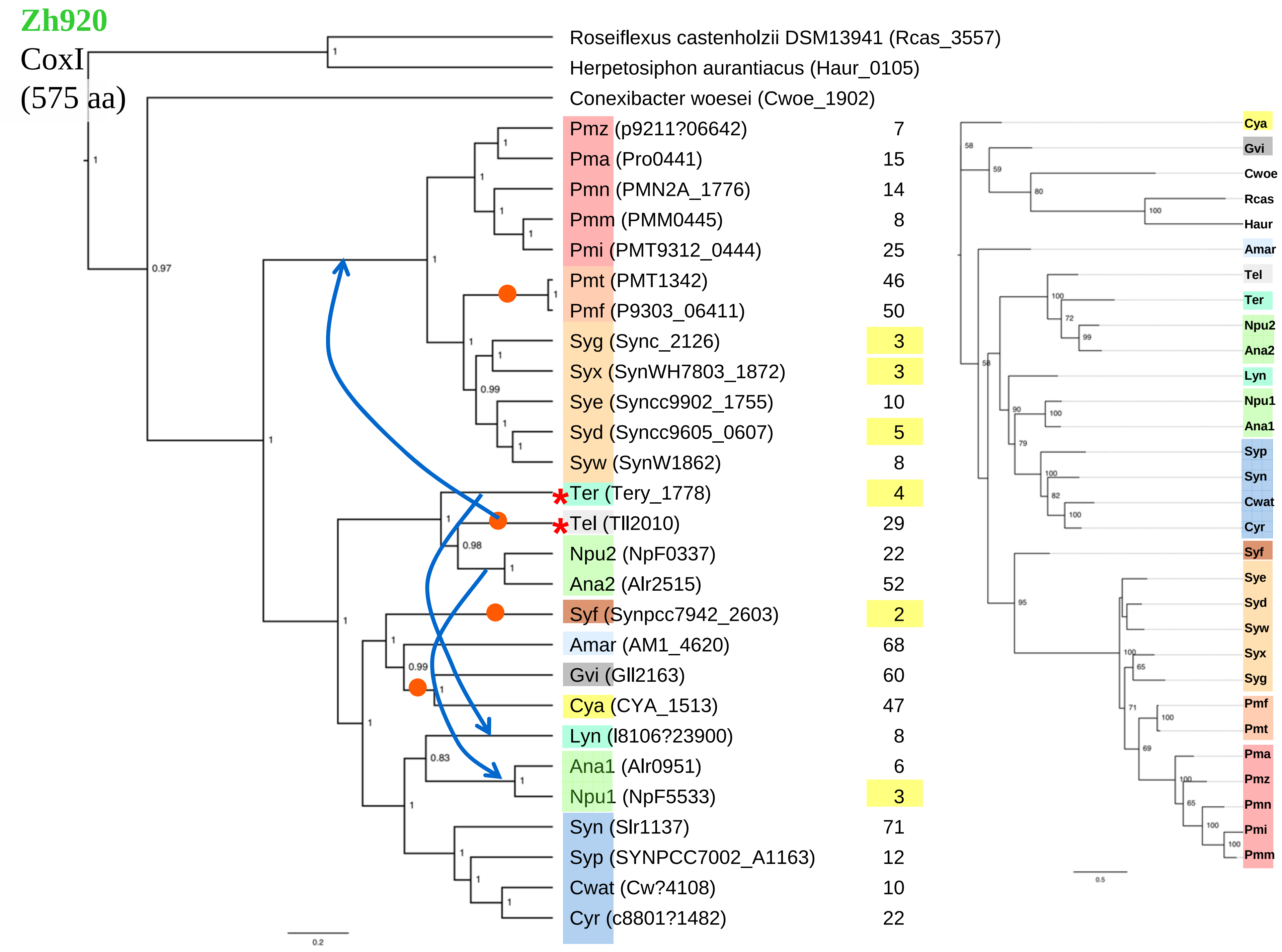
Zh920
CoxI
(575 aa)
Roseiflexus castenholzii DSM13941 (Rcas_3557)
Herpetosiphon aurantiacus (Haur_0105)
Conexibacter woesei (Cwoe_1902)
Pmz (p9211?06642) 7
Pma (Pro0441) 15
Pmn (PMN2A_1776) 14
Pmm (PMM0445) 8
Pmi (PMT9312_0444) 25
Pmt (PMT1342) 46
Pmf (P9303_06411) 50
Syg (Sync_2126) 3
Syx (SynWH7803_1872) 3
Sye (Syncc9902_1755) 10
Syd (Syncc9605_0607) 5
Syw (SynW1862) 8
Ter (Tery_1778) 4
Tel (Tll2010) 29
Npu2 (NpF0337) 22
Ana2 (Alr2515) 52
Syf (Synpcc7942_2603) 2
Amar (AM1_4620) 68
Gvi (Gll2163) 60
Cya (CYA_1513) 47
Lyn (l8106?23900) 8
Ana1 (Alr0951) 6
Npu1 (NpF5533) 3
Syn (Slr1137) 71
Syp (SYNPCC7002_A1163) 12
Cwat (Cw?4108) 10
Cyr (c8801?1482) 22
0.97
0.99
0.98
0.83
0.2
Cya
Gvi
Cwoe
Rcas
Haur
Amar
Tel
Ter
Npu2
Ana2
Lyn
Npu1
Ana1
Syp
Syn
Cwat
Cyr
Syf
Sye
Syd
Syw
Syx
Syg
Pmf
Pmt
Pma
Pmz
Pmn
Pmi
Pmm
0.5

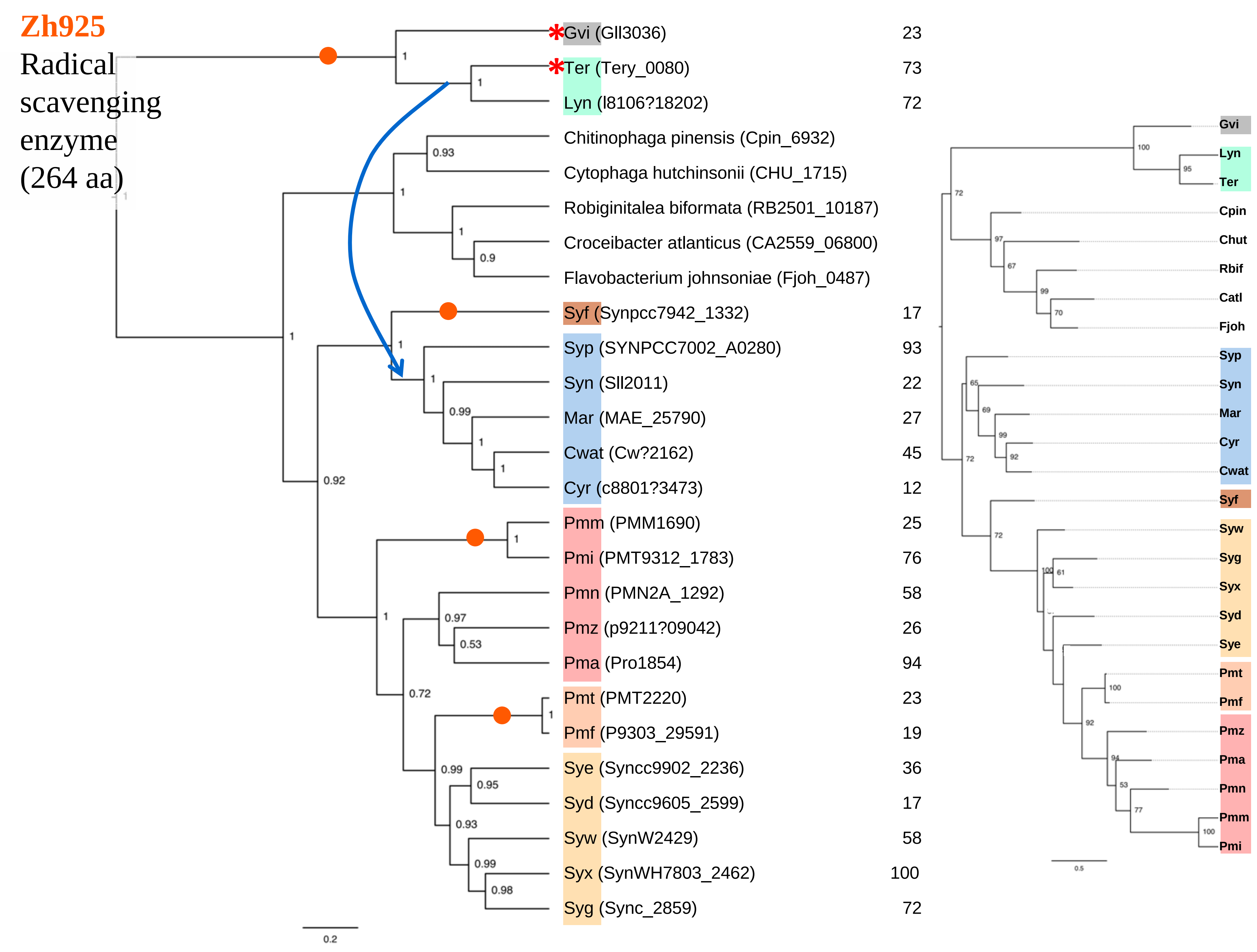

Zh925
Radical scavenging enzyme (264 aa)
Gvi (Gll3036) 23
Ter (Tery_0080) 73
Lyn (l8106?18202) 72
Chitinophaga pinensis (Cpin_6932)
Cytophaga hutchinsonii (CHU_1715)
Robiginitalea biformata (RB2501_10187)
Croceibacter atlanticus (CA2559_06800)
Flavobacterium johnsoniae (Fjoh_0487)
Syf (Synpcc7942_1332) 17
Syp (SYNPCC7002_A0280) 93
Syn (Sll2011) 22
Mar (MAE_25790) 27
Cwat (Cw?2162) 45
Cyr (c8801?3473) 12
Pmm (PMM1690) 25
Pmi (PMT9312_1783) 76
Pmn (PMN2A_1292) 58
Pmz (p9211?09042) 26
Pma (Pro1854) 94
Pmt (PMT2220) 23
Pmf (P9303_29591) 19
Sye (Syncc9902_2236) 36
Syd (Syncc9605_2599) 17
Syw (SynW2429) 58
Syx (SynWH7803_2462) 100
Syg (Sync_2859) 72
0.2
Gvi
Lyn
Ter
Cpin
Chut
Rbif
Catl
Fjoh
Syp
Syn
Mar
Cyr
Cwat
Syf
Syw
Syg
Syx
Syd
Sye
Pmt
Pmf
Pmz
Pma
Pmn
Pmm
Pmi
0.5

Zh932
ferredoxin
(118 aa)

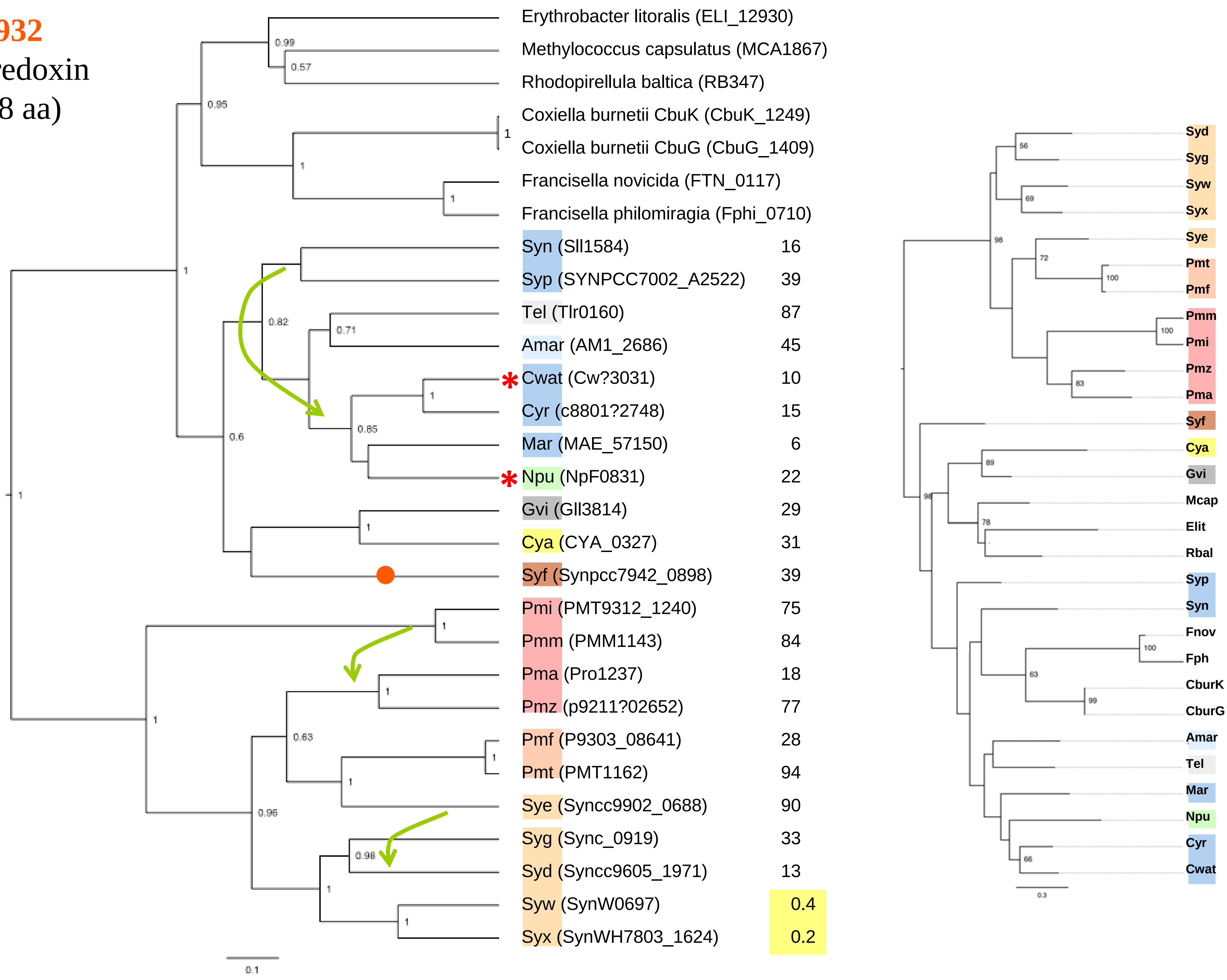

Zh934

psaL

(172 aa)

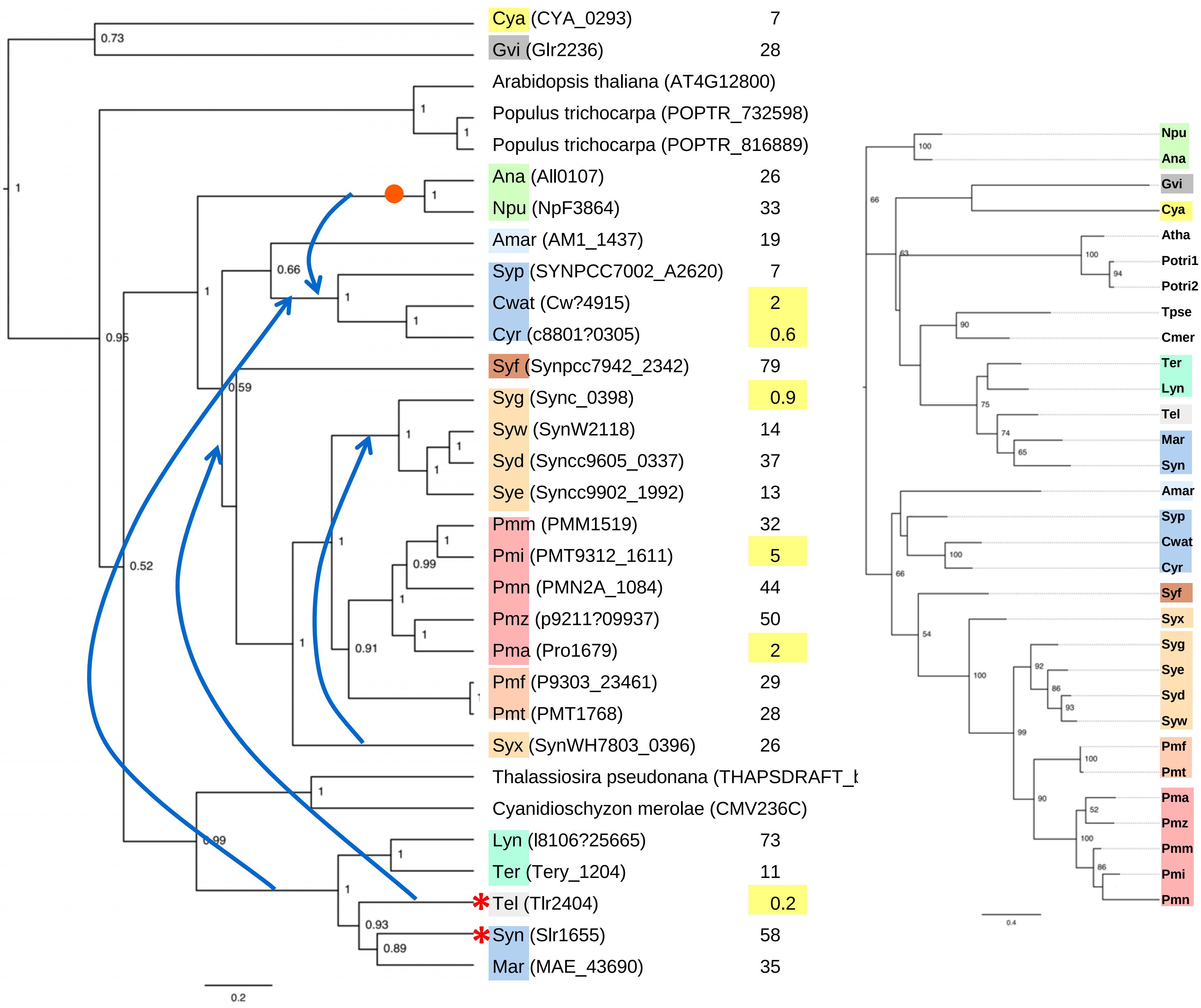

Zh986
sugar kinase
(333 aa)

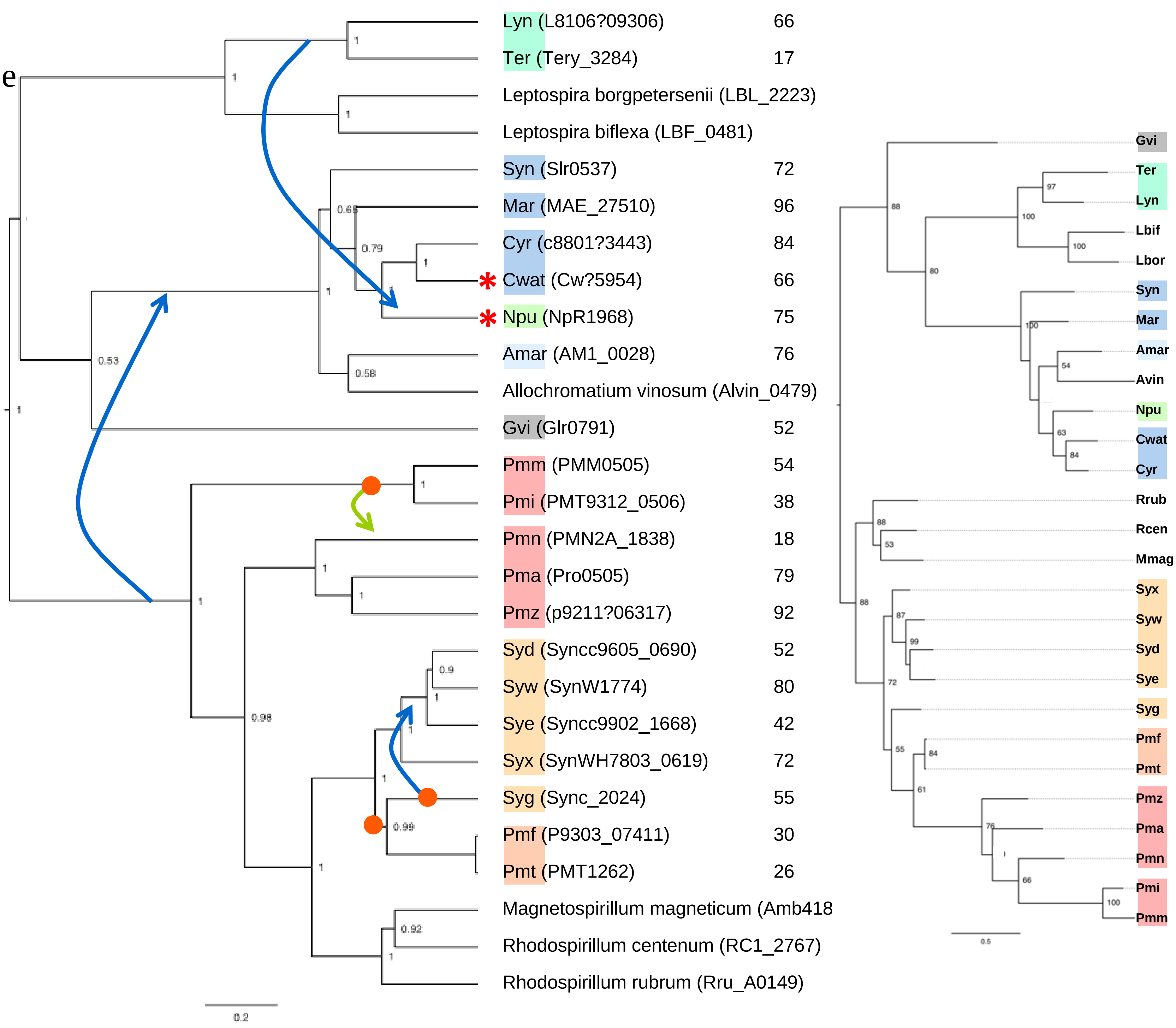

Zh1030 methyltransferase (322 aa)

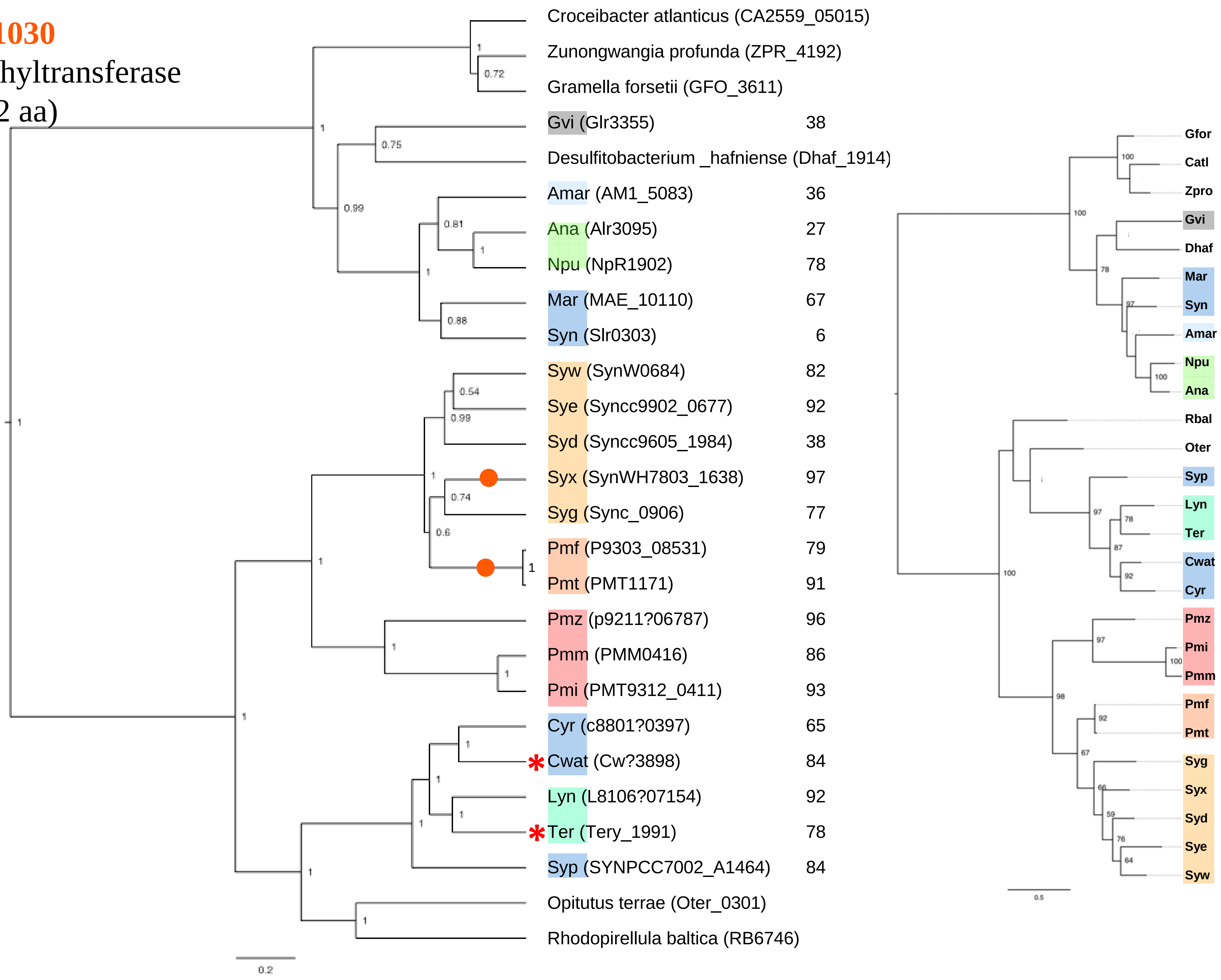

Zh1102

hypothetical

(105 aa)

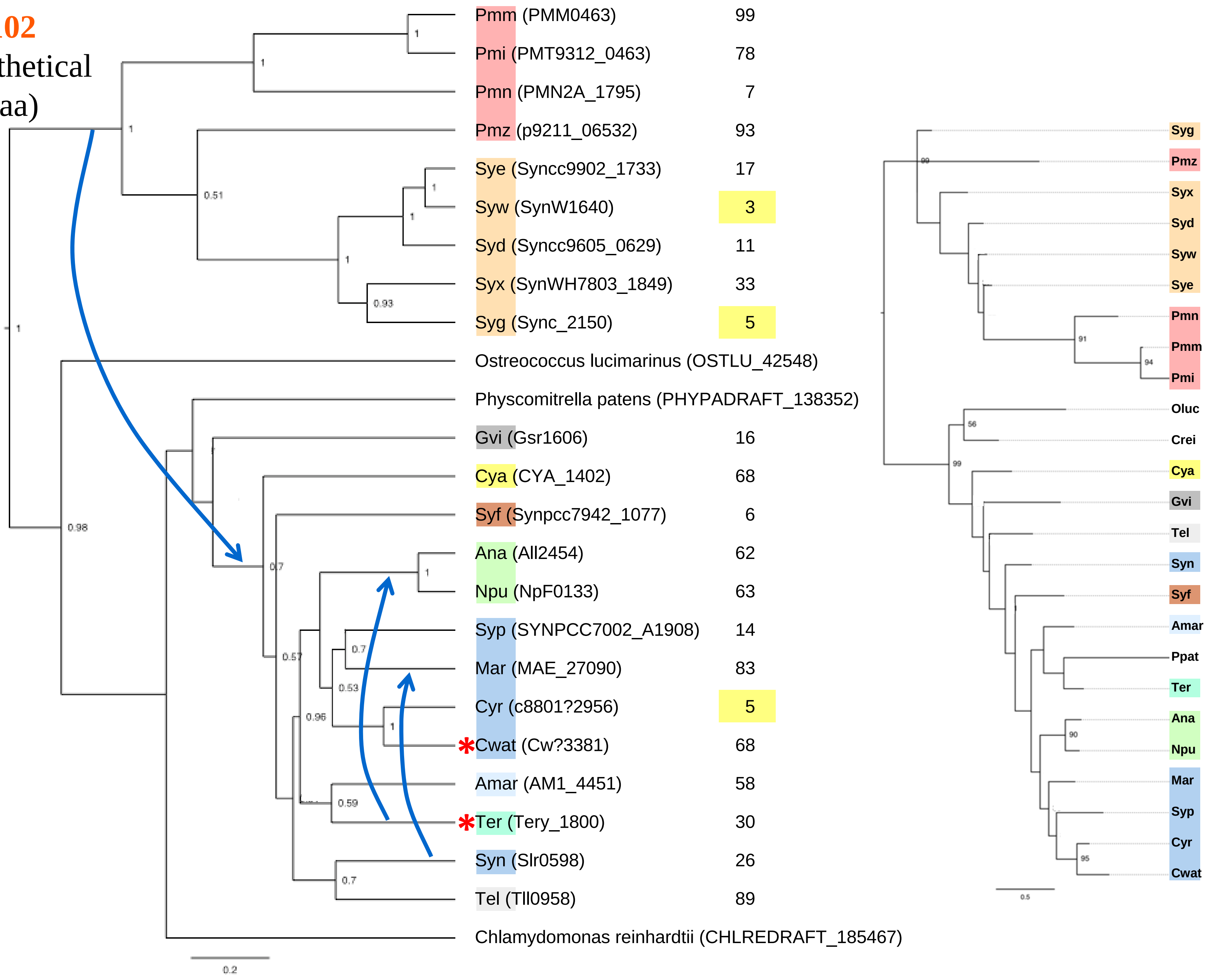

Sll1504

hypothetical (102 aa)

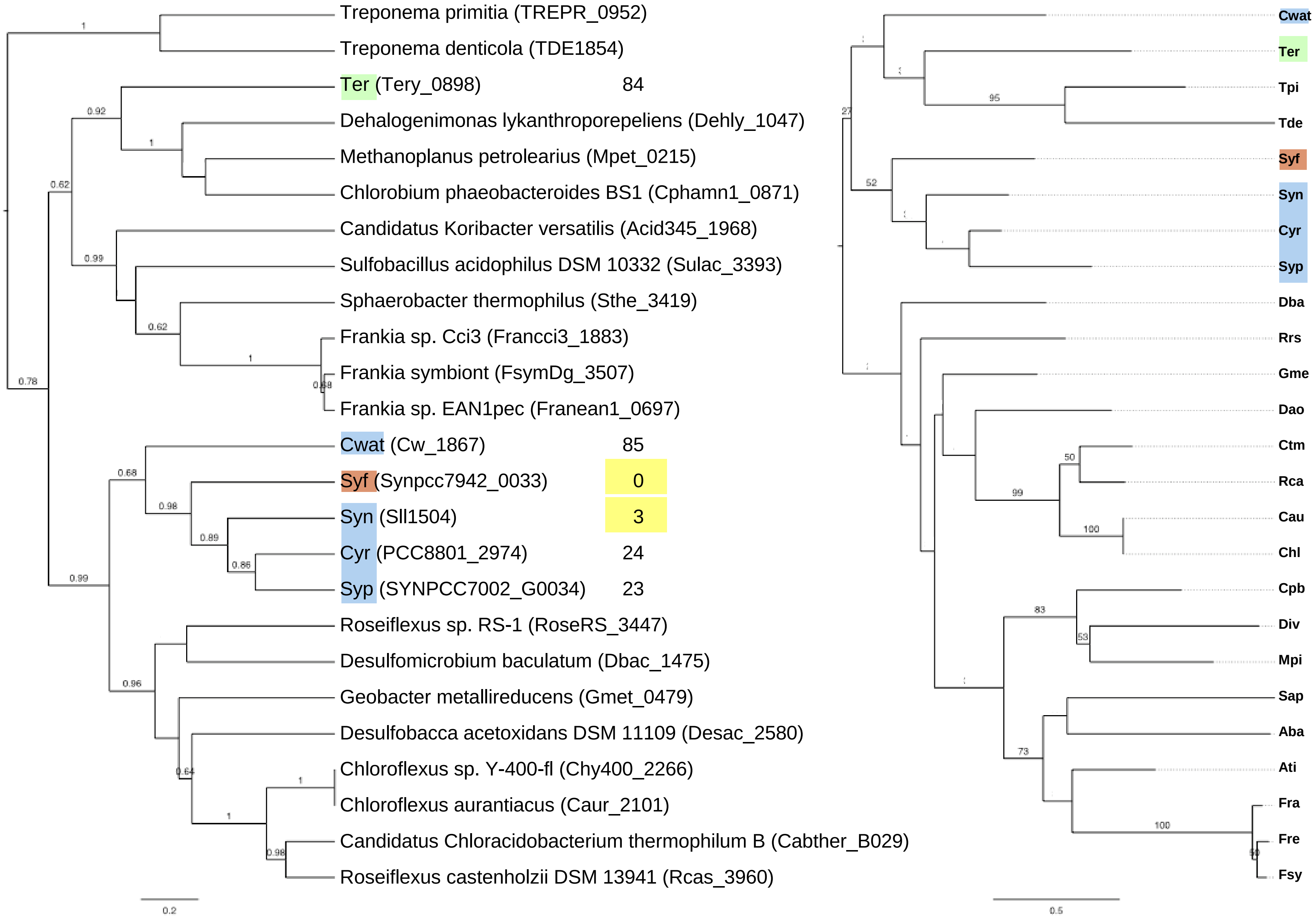

Sll1578

phycocyanin alpha subunit (162 aa)

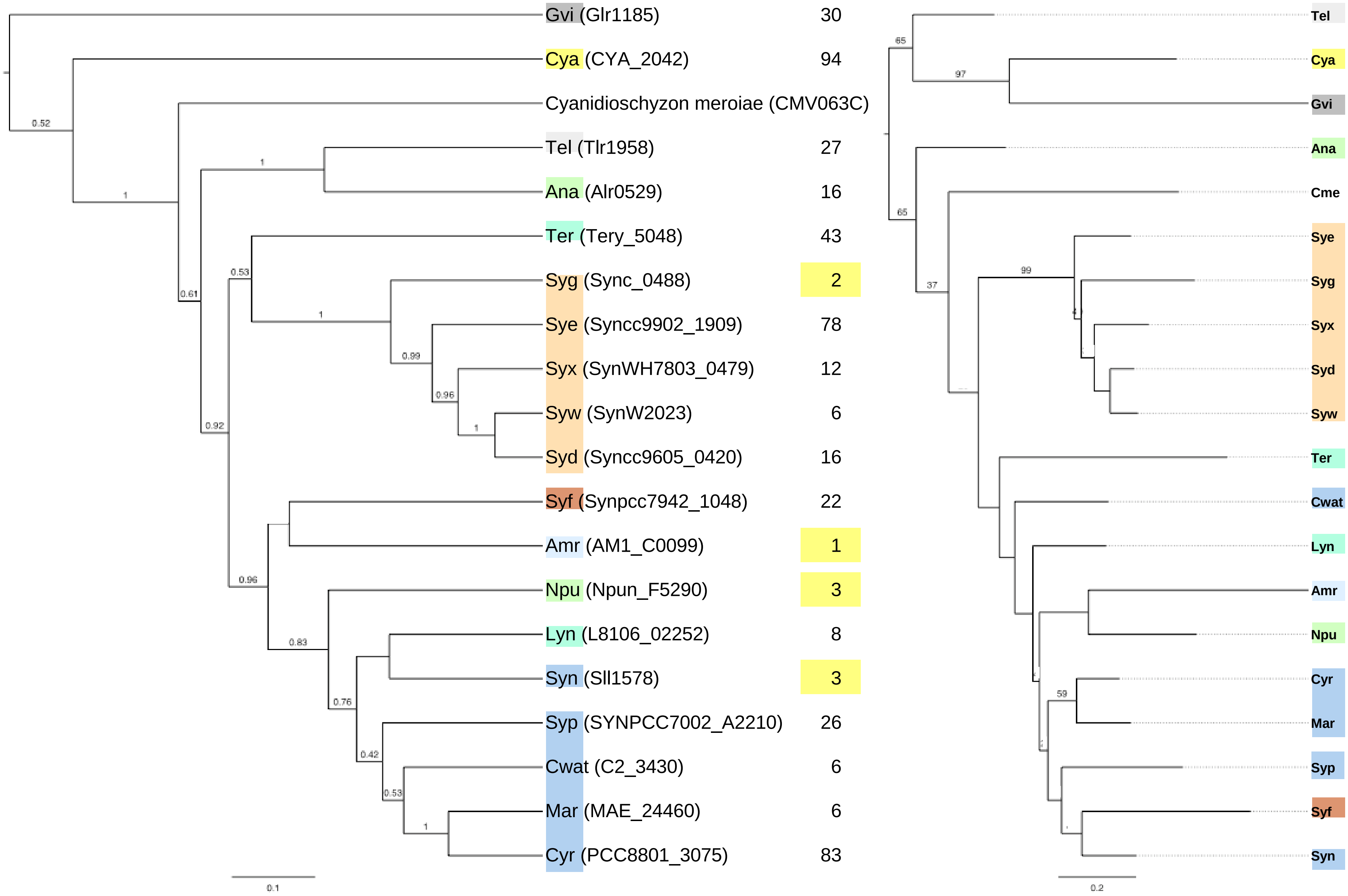

Slr1616

unknown (341 aa)

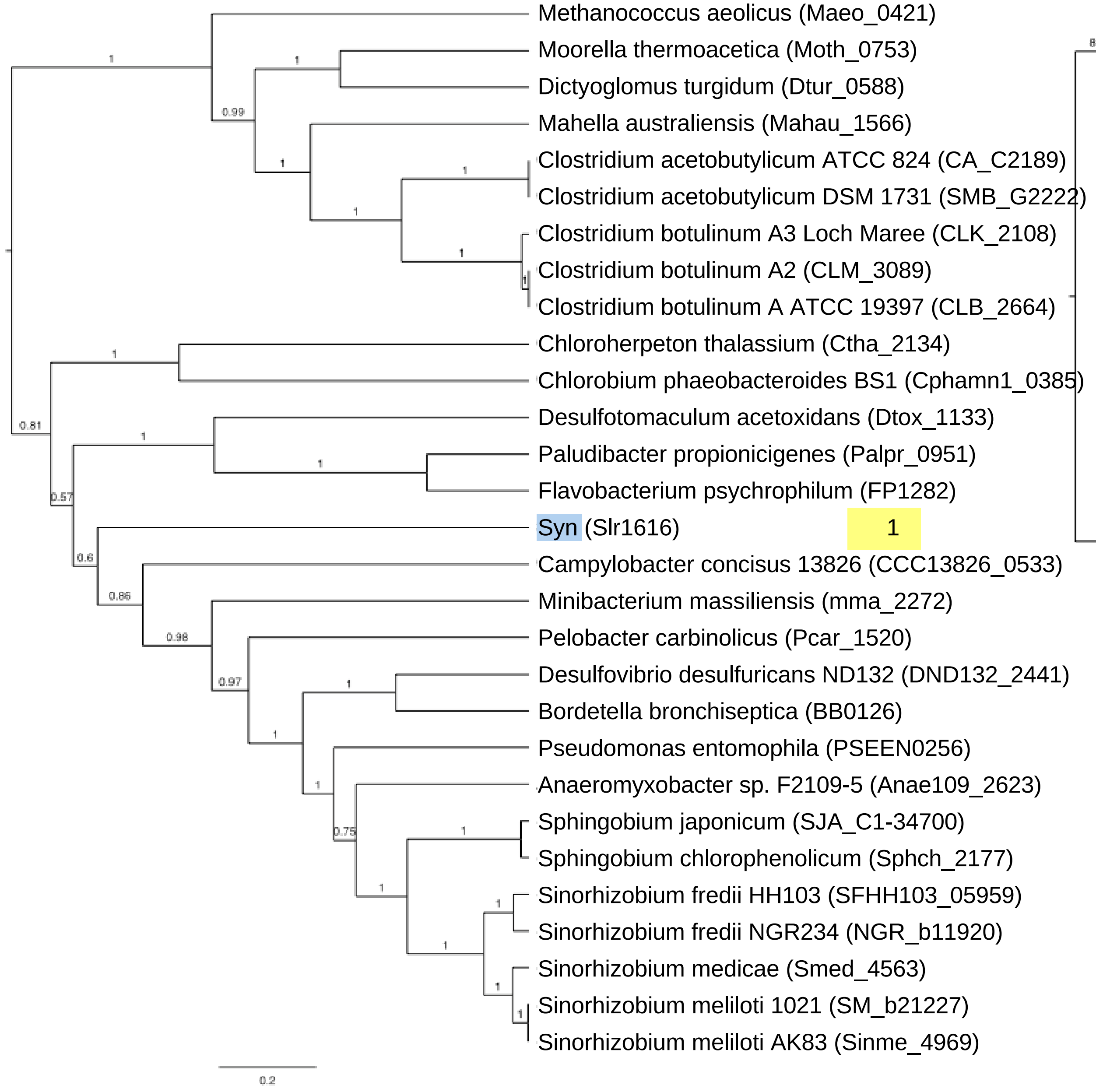

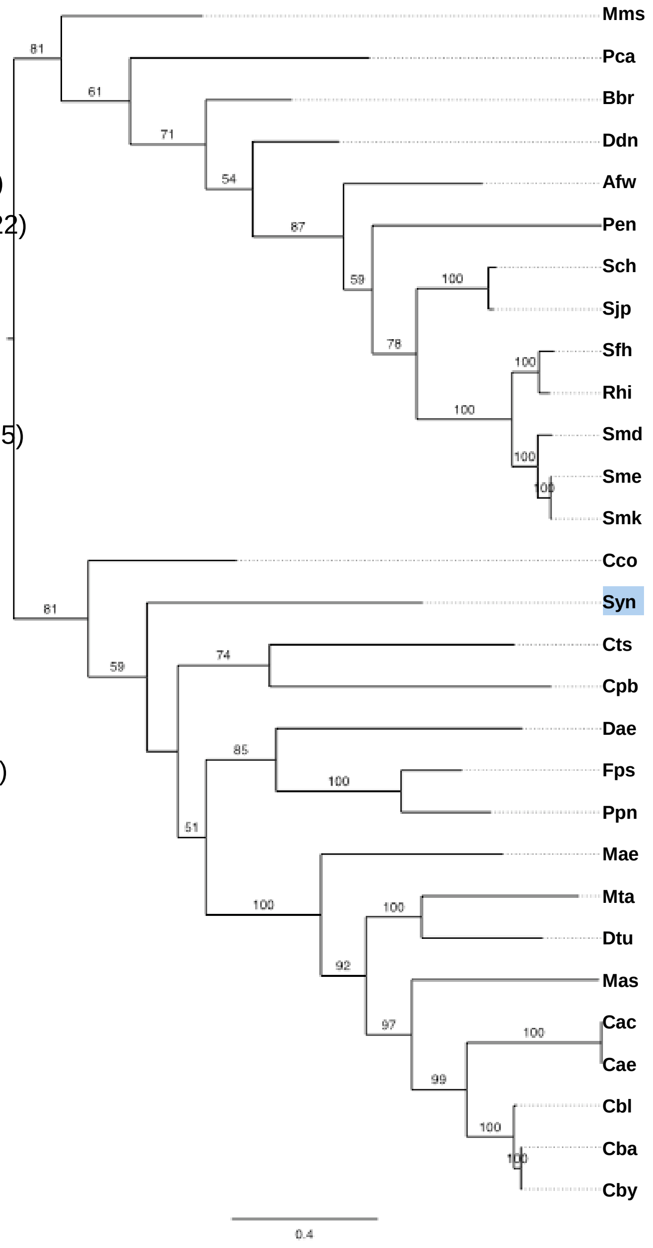

Slr0703Transposase (261 aa)

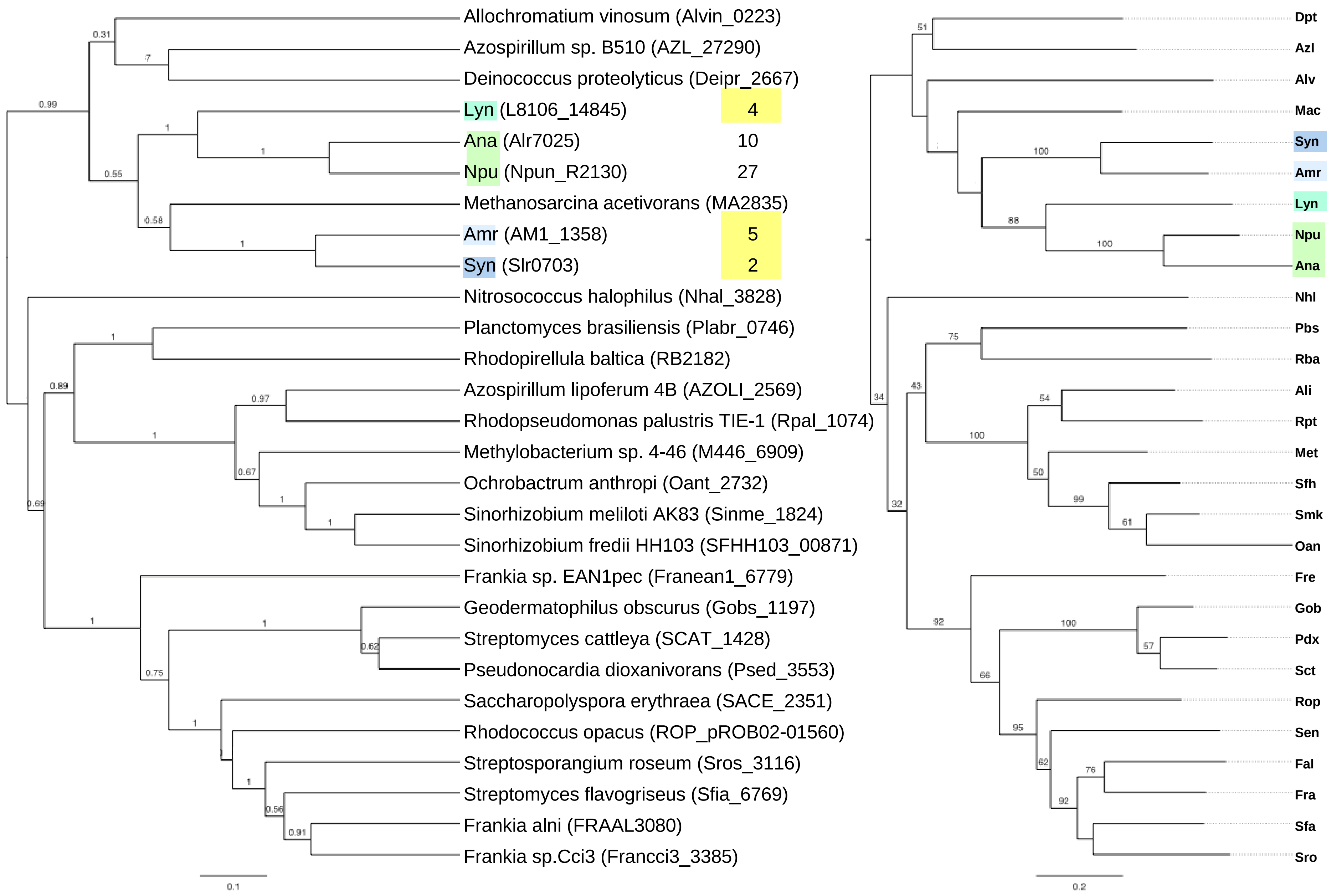

Supplement: Additional file 10 — Phylogenetic trees of conserved genes with reported conflicts. [file 1471-2164-13-245-S10.pdf]
