# Supplementary material for: Detection of horizontal transfer of individual genes by anomalous oligomer frequencies
Source: BMC Genomics. 2012 Jun 15;13:245. doi: 10.1186/1471-2164-13-245 (PMC3497702; doi:10.1186/1471-2164-13-245)
Supplement: Additional file 11 — Fraction of genes identified as putative foreign by different methods compared to CGS. [file 1471-2164-13-245-S11.pdf]

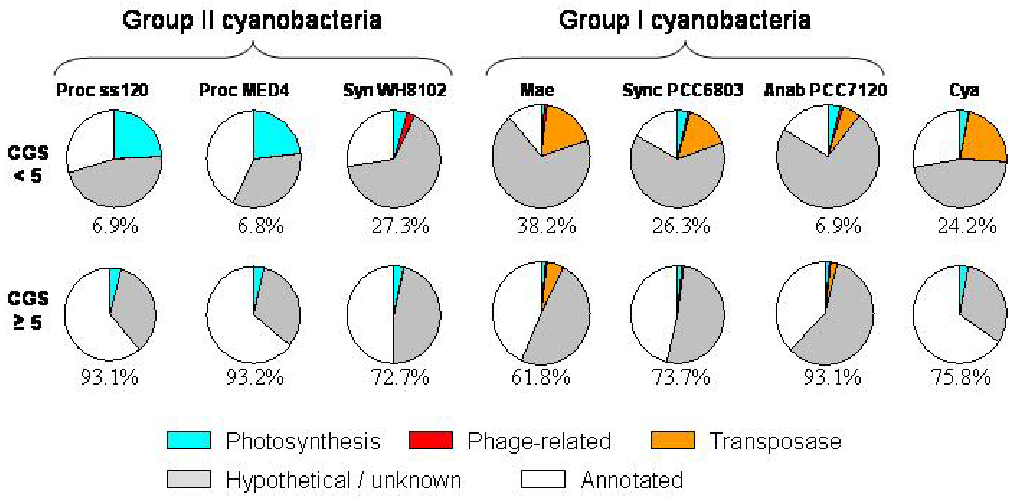

Fig. 9 – Function of genes identified as putative foreign. The distribution of genes in seven representative cyanobacteria is shown, in each case dividing the genes into two classes: those with CGS scores < 5 (top row) and those with scores ≥ 5 (bottom row). The circle represents all genes of the class.
