# Supplementary material for: Detection of horizontal transfer of individual genes by anomalous oligomer frequencies
Source: BMC Genomics. 2012 Jun 15;13:245. doi: 10.1186/1471-2164-13-245 (PMC3497702; doi:10.1186/1471-2164-13-245)
Supplement: Additional file 12 — Evolutionary context of genes of Synechocystis PCC 6803. [file 1471-2164-13-245-S12.pdf]

Additional File 10. Evolutionary context of genes of *Synechocystis* PCC 6803

		Evolutionary Context[a]			
**Genes[b]**	**N[c]**	**Cyanobacterial**	**Recent**	**Non-cyanobacterial**	**Solitary**
**Chromosome**					
CGS < 0.05	548	33%	28%	28%	11%
CGS > 0.05	2356	71%	11%	15%	3%
**Plasmids**					
CGS < 0.05	122	7%	20%	44%	28%
CGS > 0.05	157	20%	28%	36%	16%

[a] The evolutionary context describes the predominant character of the best protein matches found by Blast, particularly their similarity to proteins in related cyanobacteria. See **Methods** for details. **Cyanobacterial:** Most of the matches are to cyanobacteria and there are at least three such matches. **Recent:** Most of the matches are to cyanobacteria but there fewer than three such matches. **Non-cyanobacterial:** Most of the matches are to non-cyanobacteria. **Solitary:** The only match is to the protein itself.

[b] The protein-encoding genes of *Synechocystis* PCC 6803 were divided into to classes: those with CGS scores less than 0.05 (and thus labeled as "putative foreign") and all others.

[c] **N** represents the number of proteins in each class.
